# A Novel Method to Profile Transcripts Encoding SH2 Domains in the *Patiria miniata* Mature Egg Transcriptome

**DOI:** 10.3390/cells13221898

**Published:** 2024-11-18

**Authors:** Lauren Bates, Emily Wiseman, Alexis Whetzel, David J. Carroll

**Affiliations:** 1 Biology Professional and Transfer Programs, Southern West Virginia Community and Technical College, Logan, WV 25601, USA; lauren.bates@southernwv.edu; 2 BioSkryb Genomics, Durham, NC 27713, USA; ewiseman.phd@gmail.com; 3 Department of Biochemistry and Molecular Genetics, Midwestern University, Glendale, AZ 85308, USA; awhetz@midwestern.edu; 4 Department of Biomedical Engineering and Science, Florida Institute of Technology, Melbourne, FL 32901, USA

**Keywords:** oocyte, egg, maturation, fertilization, de novo transcriptome, annotation

## Abstract

The critical mechanism to restart zygote metabolism and prevent polyspermy during fertilization is the intracellular Ca^2+^ increase. All of the signaling molecules leading to the Ca^2+^ rise are not fully known in any species. In the sea star *Patiria miniata*, SFK1, SFK3, and PLCγ participate in this fertilization Ca^2+^ increase. These proteins share common regulatory features, including signaling via tyrosine phosphorylation and their SH2 domains. In this study, we explore two different bioinformatic strategies to identify transcripts in the *Patiria miniata* mature egg transcriptome (Accession PRJNA398668) that code for proteins possessing an SH2 domain. The first identified the longest open reading frame for each transcript and then utilized similarity searching tools to provide identities for each transcript. The second, novel, method involved a six-frame translation of the entire transcriptome to identify SH2 domain-containing proteins. The identified transcripts were aligned against the NCBI non-redundant database and the SwissProt database. Eighty-two transcripts that encoded SH2 domains were identified. Of these, 33 were only found using the novel method. This work furthers research into egg activation by providing possible target proteins for future experiments and a novel method for identifying specific proteins of interest within a de novo transcriptome.

## 1. Introduction

While *Patiria miniata* is a robust model for studying the biochemical aspects of fertilization, resources like an annotated genome, tissue-specific transcriptomes, or proteomes are not completely available, making it difficult to easily identify proteins en masse that may be functioning during fertilization. Since the 1960s, it has been well established that immature oocytes of all metazoans are transcriptionally quiescent and harbor mRNAs that are translated after fertilization to allow for rapid development [1,2,3,4,5]. The genome is not highly active until the 2-cell stage [6]. Because oocytes retain this mRNA, a sequencing database of all the mRNA in the egg (an egg transcriptome) would be a useful tool in the search for important molecules needed during egg activation and later developmental processes. While much is known about fertilization in many organisms, a complete picture of the signal transduction mechanisms that link sperm–egg interaction and fusion to the stimulation of zygote metabolism is needed. To accomplish this goal, a clear picture of the RNA and proteins expressed in the mature egg is needed. 

In this study, the first transcriptome of mature *Patiria miniata* eggs, deposited in GenBank as NCBI BioProject PRJNA398668, has been utilized to identify transcripts that code for proteins that possess at least one Src homology 2 (SH2) domain [7]. Although post-transcriptional changes during maturation do not appear to be necessary for fertilization of sea stars, the transcriptome of mature (post-germinal vesicle breakdown) *P. miniata* eggs was developed in an effort to capture any post-transcriptional changes that have yet to be detected and to avoid confusion between follicle cell transcripts and those of the mature egg. 

A fundamental and universal event at fertilization in all species that have been examined is an increase in intracellular free calcium (Ca^2+^) that is released from the endoplasmic reticulum [8]. The signaling mechanisms responsible for this Ca^2+^ increase appear to vary somewhat between different species [9]. In echinoderms, the fertilization Ca^2+^ increase results from the production of inositol trisphosphate (IP_3_) via the action of Src family kinases (SFKs) and phospholipase Cγ (PLCγ), while in mammals, the IP_3_ is produced via the action of a different phospholipase isoform, PLCζ [10,11]. The Ca^2+^ increase is necessary for important downstream egg activation events, including cortical granule exocytosis and activation of a suite of regulatory kinases [10,11]. There are almost certainly complex regulatory mechanisms needed for the transition from egg to zygote; therefore, much remains to be learned about the molecules that translate the fertilization event to the full activation of the egg. A very important first step is to identify and catalog the molecules that are present in the egg to allow for future mechanistic studies. To begin searching the transcriptome for RNAs that code for the proteins involved in egg activation [9,12], the initial focus was to identify transcripts that share an important signaling domain (Src Homology 2 or SH2 domain), which are found in the three known signaling proteins (SFK3, SFK1, and PLCγ) that function at egg activation in the sea star [13,14,15,16,17]. Src Homology 2 (SH2) domains bind phosphorylated tyrosine residues in specific peptide sequences on interacting proteins and play important roles in signal transduction in pathways involving tyrosine phosphorylation [18,19,20]. 

**Figure 1:**
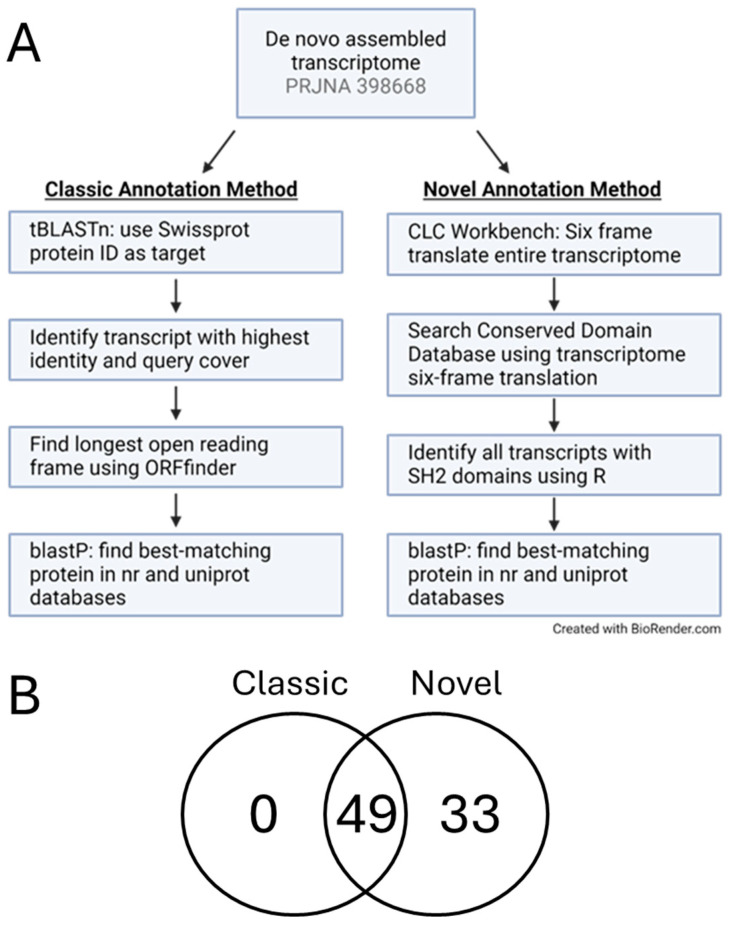
Two annotation methods were used to search the *P. miniata* mature egg transcriptome for SH2 domain-containing transcripts. (**A**) Workflow showing key differences between the methods. The classic method matches transcripts against known proteins with SH2 domains. The novel method is an unbiased method that finds all transcripts that might encode a protein that has an SH2 domain. (**B**) Both methods identified 49 transcripts that might encode a protein with an SH2 domain, while the novel method identified 33 transcripts not found using the classic method. The classic method did not identify any unique transcripts.

Two strategies were used to search for transcripts coding for SH2 domains: a “classic” method and a “novel” method, which was developed for this study (Figure 1). Both of these methods use publicly accessible databases and existing software. The classic method involves finding the longest or best open reading frame (ORFs) for each transcript using a set of software programs, including ORFfinder and Transdecoder, and aligning the ORFs against a protein database to find homologous proteins [21,22,23,24]. The “novel” method developed for this study involved first translating the transcriptome in all six frames and then searching the NCBI Conserved Domain Database (CDD), which resulted in an in silico proteome [25]. The transcripts that contained SH2 domains were then searched against the non-redundant (nr) and SwissProt databases [26] to identify homologous proteins or to potentially identify new proteins.

Through the combined analyses of both methods, 82 transcripts were identified with open reading frames that coded for a protein that contained an SH2 domain(s). Of the total, 33 were specific to the novel method (Figure 1B). Transcripts specific to the CDD search were validated using RT-PCR followed by Sanger sequencing. This study narrowed the number of potential SH2 domain-containing proteins involved in the phospho-tyrosine signaling pathway of egg activation from the original 96,723 putative transcripts to a more manageable 52, as well as contributed a new analysis pipeline for annotation of de novo assembled transcriptomes.

## 2. Methods

### 2.1. Starfish Eggs and RNA Preparation

*Patiria miniata* were collected by Marinus Scientific, LLC in Long Beach, CA, USA, and maintained in a refrigerated aquaculture system at the Florida Institute of Technology in Melbourne, FL, USA. Ovaries were collected through the dorsal epidermis of the sea star ray using a 3 mm sample corer and fine curved forceps (Fine Science Tools, Foster City, CA, USA). The ovary was minced in filtered natural seawater (FNSW) using surgical scissors, and the released oocytes were poured through 210 μm mesh to remove follicle cells and debris. These oocytes were washed three times in FNSW and, after the final wash, concentrated to a 10% solution, which is 1 mL of gravity-settled oocytes per 10 mL of seawater [27]. The washed immature oocytes (≥90% with germinal vesicle intact) were aliquoted into 1 mL samples, briefly micro-centrifuged, flash-frozen in liquid nitrogen (N_2_) after removing the excess sea water, and stored at −70 °C. Mature egg samples were produced through incubation with 1 μM 1-methyladenine (1-MA) (Fisher Scientific, Waltham, MA, USA) for 40 min to 1 h at 16 °C. Maturation was complete when the breakdown of the germinal vesicle was observed under a brightfield microscope in ≥90% of the eggs. 

Once mature, the oocytes were aliquoted into 1 mL samples of eggs in FNSW (10% *v*/*v*). For the unfertilized samples, the seawater was removed from these aliquots, and the eggs were frozen in LN_2_ and stored at −70 °C. Total RNA from the immature oocyte and egg samples was purified using the Thermo Scientific™ Gene JET purification kit (Thermo Fisher Scientific, Waltham, MA, USA) following the Mammalian Cultured Cells Total RNA Purification protocol. The RNA samples were ≥2 µg in 50 µL with A_260/280_ ranging from 2.00–2.02.

### 2.2. Library Preparation for Transcriptome Sequencing

Library preparation, sequencing, and assembly were performed by Novogene Co. (Sacramento, CA, USA). For library preparation, the NEBNext^®^ Ultra™ RNA Library Prep Kit for Illumina ^®^ (New England Biolabs, Ipswich, MA, USA) was used to create sequencing libraries from 1.5 µg of total RNA per sample following the manufacturer’s protocol, and index codes were added to the sequences in each sample. The adaptor sequences for RNA from the kit were as follows: 

5′adaptor:5′AATGATACGGCGACCACCGAGATCTACACTCTTTCCCTACACGACGCTCTTCCGATCT;

3′adaptor:3′GATCGGAAGAGCACACGTCTGAACTCCAGTCACATCACGATCTCGTATGCCGTCTTCTGCTTG. 

The PCR products generated were purified using the AMPure XP system (Beckman Coulter, Indianapolis, IN, USA), and library quality was assessed on an Agilent Bioanalyzer 2100 system (Agilent Technologies, Santa Clara, CA, USA). 

Clustering of the index-coded samples was performed with a cBot Cluster Generation System using HiSeq PE Cluster Kit cBot-HS (Illumina Inc., San Diego, CA, USA), following the manufacturer’s instructions. The clusters were sequenced on an Illumina HiSeq platform, and paired-end reads were generated.

### 2.3. Quality Assessment and Transcriptome Assembly

The raw reads in fastq format were processed through Novogene in-house Perl scripts to remove reads containing adaptors, reads containing >10% poly-N, and low-quality reads (when the base quality score, Q20, was <20 for >50% of the read). Additionally, Q20 (percentages of bases whose correct base recognition rates are >99% in total bases, Phred score ≥ 20), Q30 (percentages of bases whose correct base recognition rates are >99.9% in total bases, Phred score ≥ 30), GC content, and sequence level duplication were calculated, and downstream analysis was performed on clean data with high quality. 

Transcriptome assembly was performed by first combining the left files (read 1 file) into one left.fq, and the right files (read 2 files) were pooled into one right.fq file. These two files were assembled into the transcriptome using Trinity version r20140413p1 [24,28] with min_kmer_cov set to 2 and all other parameters set to default. Next, Corset version 1.05 [29] was used to perform hierarchical clustering to remove redundancy with a parameter set to -m 10. Finally, the longest transcripts of each cluster were selected as unigenes and assembled into one unigene.fa file. 

### 2.4. TSA Submission to NCBI

The 105,191 transcripts in unigene.fa were submitted to the National Center for Biotechnology Information’s (NCBI) Transcriptome Shotgun Assembly (TSA) database according to the TSA submission guidelines. NCBI’s contamination screen found 8468 contaminating sequences and 226 sequences to be trimmed. After removing contaminating sequences and trimming, the final TSA record submitted contains 96,723 sequences in the Bates_Pmin_unigene_edits5.fa file. The master TSA record is GGEY00000000.2 (SRA run accessions SRR6054712, SRR8926376, and SRR8926377) under BioProject PRJNA398668. 

### 2.5. Transcriptome Annotation Method 1

Two different annotation methods were used to identify transcripts that encoded SH2 domain sequences (Figure 1). For the classic annotation method, a list of proteins was generated from the literature, InterPro, and SH2db.org [30,31], which were then used as target sequences. The accession numbers corresponding to these proteins were put into NCBI tBLASTn software (Version 2.15.0) one-by-one to search against the transcriptome by choosing BioProject PRJNA398668 as the TSA database. The best matching transcript was determined by first considering if the match was ≥50% query coverage, ≥30% identity, and an e value ≤ 0.00001; however, because the entire transcript was searched against the protein of interest rather than the open reading frame, we had to consider lower scores and used the graphic summary of the results page to help determine if the best matching sequence was all assembled on one transcript or if it was possibly split onto another transcript(s). 

The resulting identified transcript(s) was further analyzed by ORFinder to identify the open reading frames (ORFs). We determined if the longest ORF or another ORF matched best to the protein of interest by searching the ORF sequence against the nr and Swissprot databases using BLASTp. An orthologous transcript was identified when it had ≥50% query coverage, ≥30% identity, and an e value ≤ 0.00001 against the protein of interest. The amino acid sequences of the transcript’s ORF and the protein of interest were analyzed using the NCBI’s Conserved Domain Database CD search to view the domains and further identify if all the domains of the protein of interest were part of the ORF of the transcript and if they were in the same arrangement. 

### 2.6. Transcriptome Annotation Method 2

To identify *Patiria miniata* mature egg transcripts that code for an SH2 domain, the transcriptome was first translated in all six frames using Qiagen CLC Main Workbench 21 (Qiagen Sciences, Germantown, MD, USA), going from 96,723 transcripts to 580,338 transcripts. Using an R script, the six-frame translated transcriptome was randomly split into files containing 4000 sequences to reduce each to a manageable size. These files were uploaded one at a time to the NCBI’s Batch CD Search tool to search against the Conserved Domain Database (CDD) using the default settings. Using R scripts, the results were combined to create a *P. miniata* mature egg conserved domain database, and any transcript with a hit for “SH2” was selected for and placed into a separate file. The transcript corresponding to a SH2 domain hit was put into ORFinder to find the open reading frames. The longest or best ORF was searched against the nr and SwissProt databases using NCBI’s BLASTp software (Version 2.15.0) to identify a homologous protein. 

### 2.7. RT-PCR

Primers were designed using NCBI’s Primer-BLAST software (Version 2.15.0) to amplify the entire ORF of transcripts of interest. Primers were prepared by Eurofins Genomics, LLC (Louisville, KY, USA). Total RNA from mature egg samples was purified using the Thermo Scientific™ Gene JET purification kit (Thermo Fisher Scientific, Waltham, MA, USA) following the Mammalian Cultured Cells Total RNA Purification protocol. The RNA samples were ≥2 µg in 50 µL with A_260/280_ ranging from 2.00 to 2.02. RT-PCR was performed using a One *Taq^®^* One Step RT-PCR kit (New England Biolabs, Inc., Ipswich, MA, USA) following the manufacturer’s protocol with annealing temperatures suggested by Eurofins Genomics. PCR products were analyzed on 0.8% agarose gels containing 100 µg/mL ethidium bromide in TAE buffer. Gels were imaged using a Bio-Rad Chemidoc XRS+ system (Bio-Rad Laboratories, Hercules, CA, USA) using the trans-UV setting. PCR products were also sent to Eurofins Genomics, LLC. for Sanger sequencing. 

## 3. Results

The *Patiria miniata* mature egg transcriptome was assembled de novo because the existing *P. miniata* genome was not fully annotated at the time of assembly, and thus alignment of the transcriptome to the genome was not informative. We utilized the latest *Patiria miniata* genome assembly, Pmin_3.0, 1 December 2020. The NCBI RefSeq assembly is GCF_015706575, and the BioProject is PRJNA597964. For annotation, the RefSeq genome records were annotated by the NCBI Eukaryotic Genome Annotation Pipeline, and proteins are predicted using the NCBI’s gene prediction software Gnomon (Genome Annotation Pipeline version 10.1) [32]. We continue to utilize the latest updates to the non-redundant protein database to identify open reading frames on each transcript. To get the most comprehensive annotation, two methods were applied to annotate the *P. miniata* mature egg transcriptome and identify any SH2 domain-coding transcripts (Figure 1). The classic annotation method for de novo assembled transcriptomes involves finding the longest open reading frame (ORF) for each transcript using ORF prediction software such as ORFfinder (RRID:SCR_016643), followed by searching the ORFs against the nr and SwissProt protein databases to identify homologous proteins. 

The second strategy is a novel method developed for this study to identify SH2 domain-containing proteins, although it could be adapted for identification of any proteins containing a specific domain. An in silico proteome was developed by translating the mature egg transcriptome in all six frames and searching the complete database against the NCBI Conserved Domain Database (CDD) (Version 3.21) [33,34]. Using a custom R script, transcripts containing SH2 domains were identified and developed as a searchable database. The identified novel SH2 domains were searched against the non-redundant (nr) and SwissProt protein databases using NCBI’s BLASTp software to identify homologous proteins or to determine if the transcript may represent a novel sequence. 

To identify SH2 domain-containing transcripts using the classic annotation method, 111 human SH2 domain-containing proteins were searched for in the transcriptome [35]. Human proteins were chosen as the targets because this is the most heavily annotated database and is expected to contain a comprehensive set of SH2-domain-containing proteins. The criteria to determine a protein match were ≥50% query coverage, ≥30% identity, e value ≤ 0.00001, and all domains of the homologous protein must be present. The classic method identified 49 transcripts that met the criteria and matched to a human orthologue. The identity of the protein coded for by each transcript was determined by alignment to the existing non-redundant protein databases, which would include *Patiria miniata* proteins identified by the automated Gnomon software. 

**Figure 2:**
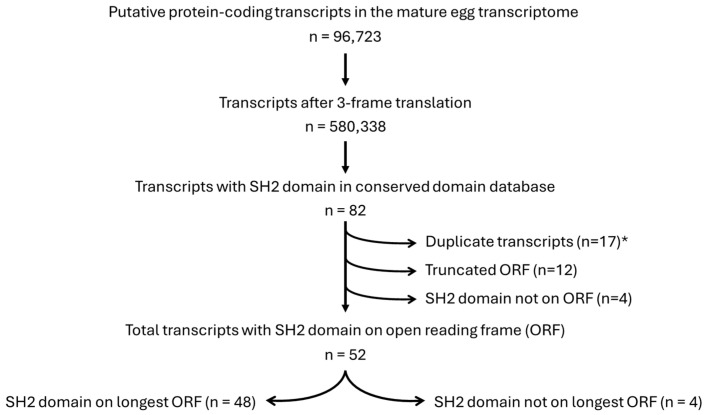
Pathway to the identification of the 52 transcripts that encode a protein with an SH2 domain contained within an open reading frame. The total number of transcripts for each step of the SH2 domain annotation process is shown above. After matching all possible transcripts (580,338) to the conserved domain database, 82 RNAs were found to encode at least one SH2 domain. Some of these were on duplicate transcripts (i.e., coded for the same amino acids), some were on truncated open reading frames (ORFs), and some were not located within an ORF, leaving 52 unique transcripts that could produce a protein containing an SH2 domain. Four of these were found to be located on an ORF that was not the largest encoded by that specific transcript. * Some of these transcripts belong to more than one category, which explains the number discrepancy (i.e., 82 − 33 ≠ 52).

The default NCBI CDD search criteria were used for the novel annotation method, and the transcripts containing SH2 domains were searched against the nr and SwissProt protein databases using the same matching criteria as for the classic annotation method. The novel annotation method also identified the 49 transcripts that were found using the classic annotation method, along with 33 additional transcripts for a total of 82 transcripts that contain SH2 domains (Figure 2 and Table S1). Taking transcripts that are duplicates, truncated ORFs, and SH2 domains not located on an ORF out of the 82 transcripts, 52 RNAs that code for a protein that contains at least one SH2 domain were identified. 

Three different broad categories of transcripts were noted using the novel method (Table 1): (1) Transcripts that matched to a known protein; (2) Transcripts with an assembly error or truncated RNA; and (3) Transcripts that did not match to a known protein via BLASTp. 

**Figure 3:**
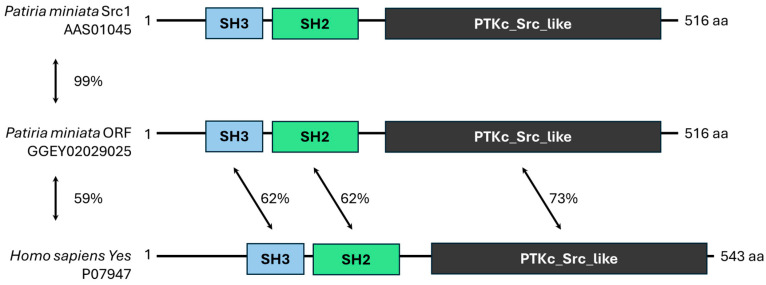
Category 1 transcript, SFK1, represents a transcript that meets all the criteria for a match to a homologous protein. The *Patiria miniata* SFK1 protein sequence (AAS01045.1) was used to identify the homologous transcript in our *P. miniata* mature egg transcriptome using NCBI’s tBLASTn software as a test of the method. The homologous transcript to AAS01045.1 was GGEY02029025.1. The top match from *Homo sapiens* in the SwissProt database was P07947, which is tyrosine-protein kinase Yes. The overall identity between the protein encoded by sea star mature egg transcriptome record GGEY02029025 and AAAS01045 was 99%, and with P07947 it was 59%. The domain arrangement is the same between the sea star and human proteins, with a 62% identity between the SH3 and SH3 domains and a 73% identity between the Src_like protein tyrosine kinase domains.

**Figure 4:**
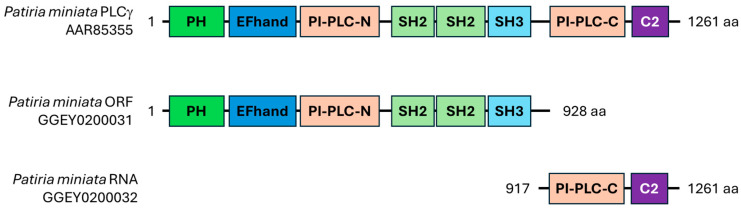
Both the classic and novel methods allow for identification of truncated transcripts. PLCγ represents a transcript that has an assembly error and shows that the expected protein is split between two transcripts. The previously identified *Patiria miniata* PLCγ protein sequence (AAR85355.1) was used to identify a homologous transcript in the *P. miniata* mature egg transcriptome using tBLASTn software of the NCBI. A) The transcripts identified as homologous to AAR85355.1 were contained within separate transcript records: GGEY02080031.1 and GGEY02080032.1. The longest ORFs of GGEY02080031.1 and GGEY02080032.1 were submitted to the NCBI CDD. GGEY02080031.1 contains all the domains of AAR85355.1 except the PI-PLC and C2 domains, which are on GGEY02080032.1. Together, these assemble into a complete PLCγ sequence. Twelve amino acids overlap between the two partial polypeptides: HERKMRIAKEFS.

**Figure 5:**
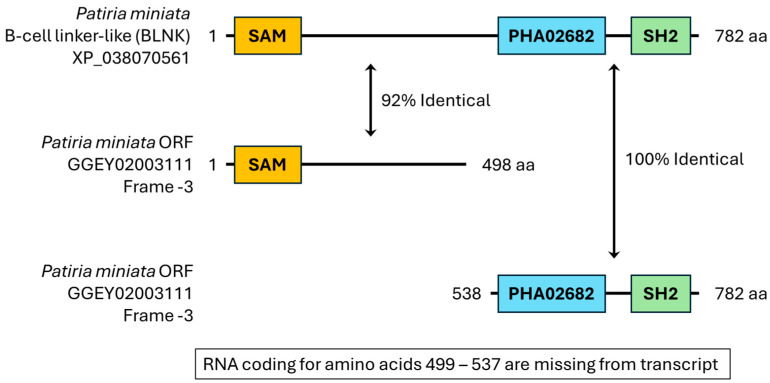
The novel method allows for identification of transcripts with assembly errors. The transcript GGEY02003111 was identified to code for an SH2 domain through the CDD search, and two proteins were identified by BLAST: extensin-like isoform X2 (XP_038070565) and B-cell linker protein-like (BLNK; XP_038070561) as possible matches. These two proteins are identical except for a missing 38 amino acid segment in XP_038070565. Shown here is BLNK aligned with the two matching frames from the transcript. The frame of GGEY02003111.1 that coded for a PHA02682 domain and a SH2 domain (frame -1) matched the identified protein at an identity level of 100%. A longer ORF on the same transcript but in frame -3 encoded a SAM domain that matched with the identified protein at an identity level of 92%. This would have been missed using the classic method.

**Figure 6:**
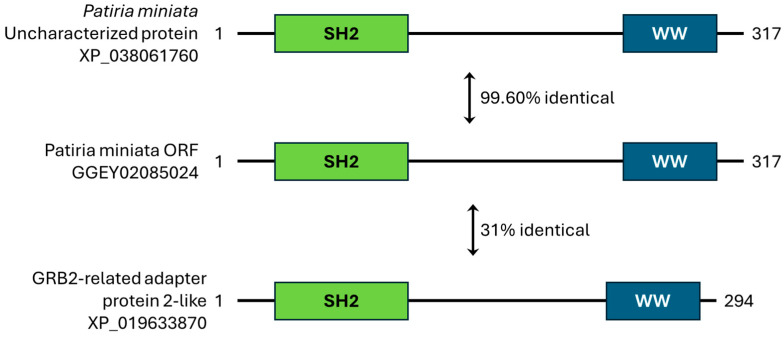
An example of category 3 transcripts, SH2/WW, represents a transcript that did not match to a known sea star protein. The ORF of the SH2/WW transcript (GGEY02085024.1) was matched against the nr and SwissProt protein databases to identify orthologous proteins using BLASTp. The best match in the nr database at 99.6% identical was to an uncharacterized *Patiria miniata* protein (XP_038061760). No characterized (known) proteins that contained the same domains (SH2 and WW) were identified using BLASTp. Identification of a protein with conserved domain architecture using CDART revealed a GRB2-related adapter protein 2-like from the lancelet *Branchiostoma belcheri*, which was 31% identical to the sea star protein at the amino acid level, with a domain architecture that matched our unidentified transcript and the uncharacterized sea star protein.

The majority of transcripts fell into the first category, with the ORF meeting the criteria of the BLASTp search, and all domains of the identified homologue were also present in the *Patiria miniata* transcript. The 49 transcripts found using both methods met these criteria (Figure 3). When a transcript met the BLASTp search criteria but did not contain the same domains and/or domain arrangement as the matching protein, it was placed into category 2. Transcripts in category 2 could either be truncated RNAs, as in the case of PLCγ (Figure 4), or they could represent assembly errors, as for an Extensin-like sequence that was identified (Figure 5). Finally, transcripts that coded for an SH2 domain but did not match any homologous protein in the databases were placed into category 3. These could be assembly errors or represent an uncharacterized protein or RNA sequence (e.g., SH2/WW, Figure 6).

**Figure 7:**
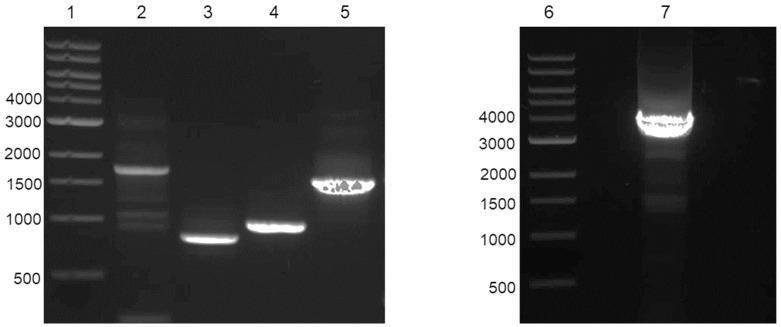
Agarose gel (0.8%) displaying RT-PCR products of the selected transcripts. A transcript(s) from each of the three categories was selected for RT-PCR to confirm the transcript assembly. Lanes 1 and 6 are the molecular weight ladders (1 kB DNA Ladder, New England BioLabs, Inc.). The remaining lanes display the RT-PCR products of SFK3 (lane 2, 1759 base pairs), Extensin/BLNK-like (lane 3, 832 base pairs), SH2/WW (lane 4, 1001 base pairs), SFK1 (lane 5, 1619 base pairs), and PLCγ (lane 7, 3754 base pairs).

To determine whether the transcripts in these categories were assembly errors or real sequences, primers were designed to amplify the full-length ORFs containing the SH2 domain of the select transcripts, and RT-PCR was performed using RNA from new mature *P. miniata* eggs. The resulting PCR products were visualized on an agarose gel and sequenced to confirm both nucleotide length and order (Figure 7). 

The transcripts selected for RT-PCR in category 1 were SFK1 and SFK3. Since these proteins are known to be involved in egg activation, they would also serve as positive controls for the assembly quality of the transcriptome. The transcripts selected to represent category 2 were PLCγ and Extensin-like, and the transcript representing category 3 was SH2/WW. Lanes 2 and 5 show clean bands representing SFK3 (1759 base pairs) and SFK1 (1619 base pairs), respectively. Both of these RT-PCR products were further confirmed with sequencing (Supplemental Figure S1). 

PLCγ is the category 2 transcript whose sequence appeared to be assembled across two transcripts (Figure 4). All protein domains were encoded in transcript GGEY02080031.1 except for two domains, which were contained in transcript GGEY02080032.1. The RNA sequences corresponding to the longest ORFs of GGEY02080031.1 and GGEY02080032.1 were combined to make one sequence, and primers were designed to amplify the entire combined sequence. The resulting RT-PCR product in lane 7 shows one large, distinct band at ~3754 base pairs, which was the predicted size for the combined sequence. When the RT-PCR product was sequenced, it was confirmed that the predicted full-length PLCγ sequence was amplified (Supplemental Figure S1) and matched to the known sequence of *P. miniata* PLCγ (AAR85355.1). 

A second transcript was amplified that also fits into category 2 is the BLNK/Extensin-like transcript, which contains the domains that are expected from B-cell linker protein-like (BLNK) (XP_038070561, Figure 5). Of note, the open reading frame coding for the SH2 domain (frame -1 in the transcript) did not contain the longest ORF. The longest ORF was on frame -3 of the transcript. When the amino acid sequences of the ORFs of these two frames were put into CD search and the domain arrangements were compared to those of XP_038070561, the longest ORF on frame -3 only matched to the SAM domain of XP_038070561, while the ORF on the -1 frame matched to the PHA03247 and SH2 superfamily domains of BLNK. To determine if frame -1 that coded for the SH2 domain was assembled correctly, primers were designed to amplify that specific region. Lane 3 of the agarose gel in Figure 7 shows the RT-PCR product of the SH2 domain-region of the BLNK/Extensin-like transcript is a single, sharp band at the correct predicted size of 832 base pairs. 

A transcript representing category 3 was SH2/WW (Figure 6). The domain comparison of the SH2/WW transcript to the top homologous proteins from the BLASTp search of both the nr and SwissProt databases shows conservation of the domain architecture. The only domain similar to the best matching proteins identified by BLASTp was the SH2 domain, and the best matching homologous proteins also did not meet the criteria for determining a match (Query cover of 50% and identity >30%), thus this transcript was initially thought to represent a unique sequence. Primers were designed to amplify the longest ORF of the SH2/WW transcript (GGEY02085024.1), which contained the SH2 domain. The RT-PCR product is represented in lane 4 of the agarose gel in Figure 7. The RT-PCR produced a single band at ~1001 base pairs, which was the predicted fragment size. The sequence of the RT-PCR product was further confirmed with sequencing (Supplemental Figure S1). However, analysis of the SH2/WW transcript using the conserved domain architecture retrieval tool (CDART) [36] of the conserved domain database revealed a possible matching protein GRB2-related adapter protein 2-like from *Branchiostoma belcheri.* The sequence conservation at the amino acid level is only 31%, which results in this protein not being found using BLASTp. However, the domain architecture is conserved between these proteins (Figure 6). 

## 4. Discussion

### 4.1. Development of Novel De Novo Transcriptome Annotation Strategy

The primary goal of this study is to build a database of RNAs that could code for signaling proteins that function at fertilization and/or early development. This is particularly difficult in model systems, like echinoderms, for which established molecular tools do not readily exist. Identifying these transcripts represents the first step in building some of these tools (antisense RNAs, fusion proteins, antibodies, etc.) for use in mechanistic studies. There are two major contributions from this study: (1) a novel annotation method for de novo transcriptomes, and (2) the identity of potential SH2 coding transcripts in the *Patiria miniata* mature egg. During annotation of the *P. miniata* egg transcriptome, the CDD search identified 33 further transcripts coding for SH2 domains that include transcripts with assembly errors and new transcripts that were missed using the classic annotation strategy, which identified 49 transcripts. A total of 82 transcripts were identified in the *P. miniata* mature egg transcriptome that coded for a protein with at least one SH2 domain. After accounting for duplicates (RNAs that code for the same protein), truncated transcripts, and SH2 domains that did not appear to be a part of a larger protein, the final count of RNAs was 52 (Figure 2). 

Many researchers working with de novo assembled transcriptomes are interested in identifying transcripts that are homologous to well-characterized proteins with the goal of identifying proteins that are present in that cell at that point in time. The classic annotation strategy is to identify the longest ORFs (using software like ORFfinder or Transdecoder), as they most likely represent the protein coding sequence, and align these ORFs to well-annotated protein databases (using software like BLASTp against the NCBI nr and SwissProt databases) [25,37,38,39,40]. While this strategy does identify many transcripts, it is limited in its ability to detect assembly errors and would not typically identify transcripts coding for novel proteins or for orthologues with a low identity at the amino acid level. 

The novel annotation method developed here offers increased breadth for the annotation of de novo assembled transcriptomes because it searches all six frames of translation for each transcript against the NCBI CDD database. Searching initially for a specific domain, rather than the entire protein, provides the possibility of identifying orthologues with lower overall amino acid sequence identity, assembly errors, and/or transcripts that code for novel proteins [41]. The Annocript pipeline also searches the CDD database; however, its purpose is to identify long non-coding RNAs, not new proteins [41,42,43,44]. 

### 4.2. Transcripts Identified Using the Novel Annotation Method

#### 4.2.1. Extensin/BLNK-like 

The Extensin/BLNK-like transcript was encoded on a single RNA transcript but not within a single open reading frame (ORF). This appears to be an assembly error in which the N-terminus of the protein (amino acids 1–475), including the SAM domain, was encoded in frame -3 of the transcript, while the C-terminus (amino acids 515–759) was encoded in frame -1. This also resulted in the codons for amino acids 476–514 missing from the transcript. The RNA does appear to be present in the mature egg, as demonstrated by amplification of an 832 base pair sequence against the ORF containing the SH2 domain (Figure 5). 

Extensins are a family of hydroxyproline-rich glycoproteins that reside in the cell walls of plants, lack domains, but have certain motifs, and play major roles in plant development and defense [43]. The Extensin-like protein, XP_038070565, that matched the *P. miniata* transcript did not resemble typical Extensin proteins because it contained several domains. In addition to the SH2 domain, the Extensin-like protein match contains a PHA02682 domain identified as an Orf virion core protein domain [45]. Additionally, a SAM superfamily domain resides at the N-terminus of this BLNK, which is a helical protein domain involved in protein interactions in bacteria and eukaryotes [46]. BLNK is a cytoplasmic adaptor protein that is an essential part of the signalosome in B cell receptor activation—which includes protein tyrosine kinases Lyn, Syk, and Btk, and BLNK, PLCγ2, PI3K—and is necessary for pre-B cell to pro-B cell transition and B cell apoptosis [47]. 

When reviewing the multiple sequence alignment of the Extensin-like protein, the BLNK-like protein, and the *P. miniata* transcript, the highest identity is in the SH2 domain. While the sequence was validated with RT-PCR and Sanger sequencing, the protein product of this transcript has yet to be identified in the *P. miniata* mature egg, and if present, the function of this protein will need to be assessed. Based on the homologous protein function and domain analysis, it is likely this transcript codes for a protein that acts as an adaptor or membrane linker involved in tyrosine kinase signaling. 

#### 4.2.2. SH2/WW

The initial characterization of this transcript suggested a possible assembly error, as it represents a fragment of a larger transcript. However, amplification of the entire ORF coding for this transcript by RT-PCR using freshly isolated egg mRNA demonstrated that it was not an assembly error but was indeed a real sequence of the expected size of 951 base pairs that could code for a 317 amino acid protein (Figure 6 and Figure 7). 

WW domains are small domains of approximately 40 amino acids and contain two conserved tryptophan (W) residues separated by ~28–30 amino acids [48,49]. WW domains function in many signaling pathways, binding to phospho-serine or phospho-threonine on interacting proteins [33]. This would be the first identification of a protein containing only a WW and SH2 domain in echinoderms. This protein could, perhaps, be an important adaptor to bridge and simultaneously transduce a signal for tyrosine and serine/threonine phosphorylation signaling pathways that are necessary for egg activation. However, before this theory can be tested, it is imperative to determine if there is a protein product for this transcript in the mature egg. 

### 4.3. Comparison of SH2 Domain-Containing Transcripts Found in Sea Star Egg Transcriptome to SH2 Domain-Containing Proteins Involved in T Cell Receptor Activation Signaling

The second major contribution of this research is the identification of potential SH2 domain-coding transcripts in the *P. miniata* mature egg to provide target molecules for understanding signaling at fertilization. SH2 domains are responsible for binding to phosphorylated tyrosine residues on interacting proteins and play a major role in transducing signaling in pathways controlled by tyrosine phosphorylation. The three proteins known to be involved in sea star egg activation (SFK1, SFK3, and PLCγ) all signal via SH2 domains. Therefore, identifying transcripts with SH2 domain coding potential should provide more protein targets for investigation into a role in egg activation. Initially, there were 82 transcripts in total found that code for a protein that contains an SH2 domain. Transcripts that had the same ORF coding for the same SH2 domain-containing protein were counted as one identity, which brought the total number of unique SH2 domain-coding ORFs in the mature starfish egg to 52. The functional category of the proteins identified in this study includes adaptor proteins, kinases, phosphatases, guanine-nucleotide exchange factors, ubiquitin ligase, a lipase, signal transducer and activator of transcription-like (STAT) proteins, suppressor of cytokine signaling-like (SOCS) proteins, transcription elongation factor SPT6-like, and CATSPER1. There were three transcripts that encoded uncharacterized proteins and four transcripts that only matched the SH2 domain superfamily. 

The signaling pathway leading to Ca^2+^ release in the sea star egg can be modeled after the pathway leading to internal Ca^2+^ release during T cell activation. When a T cell receptor (TCR) engages with a peptide in a major histocompatibility complex (pMHC) on an antigen presenting cell (APC), the immunoreceptor tyrosine-based activation motifs (ITAMs) on the TCR are phosphorylated by two Src family kinases, Lck and Fyn [49,50]. The phosphorylated ITAMs recruit and activate the tyrosine kinase ζ-chain associated protein of 70 kDa (ZAP-70). ZAP-70 promotes the formation of protein microclusters, which are hubs for signaling complexes to form and are important for propagation of signaling pathways necessary for TCR activation [51,52]. One of these is the pathway to intracellular Ca^2+^ release from the endoplasmic reticulum. 

The microcluster promoting intracellular Ca^2+^ release consists of a collection of adaptor proteins, kinases, and guanine nucleotide exchange factors (GEFs), including: Linker for Activation of T cells (LAT), SH2 domain containing leukocyte protein of 76 kDa (SLP-76), Vav1, Itk, c-Cbl, PLCγ1, Grb2, and Grb2 related adaptor protein (GADS) [45,46,47,48,49]. Through the formation of this microcluster, PLCγ1 is phosphorylated and activated, leading to an intracellular Ca^2+^ increase. The increase in Ca^2+^ leads to processes in cytotoxic T cells like increased motility and lysis of infected target cells and processes in helper T cells (Th) like cytokine production and differentiation of naïve T cells into Th1, Th2, or Th17 cells [53,54]. 

Of the proteins involved in the microcluster, ten of them signal through SH2 domains, including: Lck, Fyn, ZAP-70, SLP-76, Itk, PLCγ1, Grb2, GADS, Vav1, and c-Cbl. Since the pathway to Ca^2+^ release in egg activation is similar to that in T cell activation [54], it made sense to determine if any of the transcripts with SH2 domains in the sea star egg transcriptome had homology to the T cell activation proteins with SH2 domains. In the sea star egg, the homologous proteins identified were Grb2, PLCγ, and Cbl. Although the protein tyrosine kinases did not match exactly, there were twelve tyrosine kinases identified in the egg transcriptome, including SFK1 and SFK3, which we know are required for Ca^2+^ release at egg activation in the sea star [55,56], in addition to Csk, Syk, Yes, JAK2, HTK16, Fer, Csk, Src42A, Abl, myosinIIIb-like, and BMX. 

The novel annotation method identified 33 more transcripts than the traditional method. While this data greatly narrowed down targets to explore in functional studies, because the egg houses mRNAs for later developmental processes, it is imperative to determine which transcripts have a protein product in the mature egg before functional studies are performed [57]. 

The major outcomes from this analysis include a searchable transcriptome library for proteomics data, the identification of assembly errors in the mature egg transcriptome, and the development of a library of SH2 domain targets for use in future proteomic studies to understand the process of fertilization. The accidental observation of the fragmented PLCγ protein illuminated these errors, which will improve the alignment of proteomics data to the transcriptome, primarily for investigating hits that may have low identity scores so that matches below a certain threshold will not be immediately eliminated. The SH2 domain analysis has also provided exciting new information in regard to potential new proteins that could be functioning in the egg, whether at the moment of fertilization or in a later developmental process. 

## Figures and Tables

**Table 1:** Classes of transcripts identified using both annotation strategies.

Classes of Transcripts Found	Example Transcript
(1) Transcript matched to known protein	GGEY02029025.1_+2 matched to AAS01045.1; Src family tyrosine kinase [*Patiria miniata*]
	GGEY02062432.1_-2 matched to AAS01047.1; Src family kinase [*Patiria miniata*]
(2) Transcript assembly error or truncated RNA	Assembly error: GGEY02003111.1_-1 matched to XP_038070565: Extensin-like isoform X2 [*Patiria miniata*]
	Truncated RNA: GGEY02080031.1_+2 and GGEY02080032.1_-3 matched to AAR85355.1; phospholipase C-gamma [*Patiria miniata*]
(3) Transcript did not match to a known protein via BLASTp	GGEY02085024.1_+2 matched to XP_022098870.1; uncharacterized protein LOC110983703SH2 (SH2+WW)

## Data Availability

Data used for the analysis in this manuscript is available on the National Center for Biotechnology website of the National Institutes of Health. The BioProject Accession number is PRJNA398668.

## Supplementary Materials

The following supporting information can be downloaded at: https://www.mdpi.com/article/10.3390/cells13221898/s1, Figure S1: Sanger sequencing results of amplified transcripts aligned to the respective transcriptome sequences; Table S1: SH2 domain containing transcripts identified and the homologous protein matches.
