# Supplementary material for: A Novel Method to Profile Transcripts Encoding SH2 Domains in the Patiria miniata Mature Egg Transcriptome
Source: Cells. 2024 Nov 18;13(22):1898. doi: 10.3390/cells13221898 (PMC11593052; doi:10.3390/cells13221898)
Supplement: Supplementary file 1 [file cells-13-01898-s001.zip › cells-3249580-supplementary.pdf]

Supplemental Material

Table S1. SH2 domain containing transcripts identified and the homologous protein matches. The homologous protein matches met the following criteria unless otherwise noted ≥50% query coverage, ≥30% identity, e value ≤0.00001. The "+" or "-" following the transcript ID number indicates the coding frame of the SH2 domain in that RNA.

**Protein Identity organized by functional category**	**Orthologue**	***P. miniata* transcript**	**Query Cover**	**Percent Identity**	**E value**
		**Adaptors**			
adapter molecule Crk-like (CRK)	XP_022099500.1	GGEY02028530.1_-2	94%	80.89%	0
		GGEY02028531.1_-2			
cytoplasmic protein NCK2-like isoform X3 (NCK2)	XP_022098621.1	GGEY02027870.1_+1	100%	85.79%	0
GRB2-related adaptor protein 2-like isoform X2 (GRAP2)	XP_022080390.1	GGEY02021954.1_-2	100%	79.38%	7e-150
		GGEY02002088.1_-1	99%	77.12%	4e-59
growth factor receptor-bound protein 14-like isoform X2 (GRB14)	XP_022096619.1	GGEY02070764.1_+3	100%	84.42%	0
		GGEY02070765.1_+2			
		GGEY02070766.1_+1			
growth factor receptor-bound protein 2-like isoform X1 (GRB2)	XP_022107052.1	GGEY02025092.1_+3	99%	69.59%	2e-101
		GGEY02025093.1_+3	99%	70.05%	4e-102
growth factor receptor-bound protein 2-like isoform X2 (GRB2)	XP_022080350.1	GGEY02086015.1_+2	100%	84.05%	2e-143
phosphatidylinositol 3-kinase regulatory subunit alpha-like isoform X1 (PIK3R1)	XP_022087778.1	GGEY02075311.1_+2	100%	88.62%	0
SH2 domain-containing adapter protein F-like isoform X4 (SHF)	XP_022104660.1	GGEY02080172.1_+1	100%	82.57%	0
		GGEY02083465.1_+1	97%	82.59%	2e-173
SH2 domain-containing protein 3C-like isoform X2 (SH2D3C)	XP_022100995.1	GGEY02012666.1_+3	100%	83.58%	0
		GGEY02012669.1_+2	100%	88.27%	0
		GGEY02012670.1_+3			

SH2 domain-containing protein 4B<br><br>(SH2D4B)	XP_022087420.1	GGEY02081351.1_-2	100%	82.80%	0
SH2B adapter protein 1-like isoform X1<br><br>(SH2B1)	XP_022096203.1	GGEY02082959.1_+1	100%	80.08%	0
SH3 domain-binding protein 2<br><br>(SH3BP2)	XP_022086558.1	GGEY02088955.1_+2<br>GGEY02088956.1_+2	96%	57.97%	1e-44
SHC-transforming protein 3<br><br>(SHC3)<br><br>*ID chosen based on best matching domains	Q61120.2	GGEY02058980.1_+2	93%	39.18%	3e-101
Signal-transducing adaptor protein 2<br><br>(STAP2)	Q9UGK3.2	GGEY02055296.1_-2	68%	22.85%	7e-11
		GGEY02057341.1_-2	67%	22.85%	5e-11
**Kinases**					
C-terminal Src kinase<br><br>(Csk)	AAS01044.1	GGEY02090463.1_-1	97%	100%	0
cytoplasmic tyrosine-protein kinase BMX-like<br><br>(BMX)	XP_022084812.1	GGEY02057172.1_-3<br>GGEY02057402.1_-2	100%	78.13%	0
myosin-IIIb-like isoform X2	XP_022097396.1	GGEY02055549.1_+1	100%	87.44%	0
Src family kinase<br><br>(SRC)	AAS01047.1	GGEY02062432.1_-2	100%	99.29%	0
Src family tyrosine kinase<br><br>(YES1)	AAS01045.1	GGEY02029025.1_+2	100%	99.81%	0
tyrosine-protein kinase ABL1-like isoform X3<br><br>(ABL1)	XP_022106292.1	GGEY02034428.1_+2	100%	90.71%	0
Tyrosine-protein kinase Src42A<br><br>(Src42A)	Q9V9J3.1	GGEY02051612.1_+1	47%	60.81%	0
tyrosine-protein kinase CSK-like<br><br>(CSK)	XP_022100836.1	GGEY02022650.1_-2	99%	80.25%	0
tyrosine-protein kinase Fer-like isoform X2<br><br>(FER)	XP_022100486.1	GGEY02018068.1_-2	96%	76.65%	0
tyrosine-protein kinase HTK16-like	XP_022088083.1	GGEY02054522.1_-1	98%	89.80%	0
		GGEY02063425.1_-2	100%	86.98%	0

(HTK16)					
tyrosine-protein kinase JAK2-like<br><br>(JAK2)	XP_022109093.1	GGEY02037849.1_-3<br>GGEY02060004.1_-3	100%	74.05%	0
tyrosine-protein kinase SRK2-like<br><br>(YES1)	XP_022088016.1	GGEY02081616.1_-1<br>GGEY02052567.1_-1<br>GGEY02003261.1_-1<br>GGEY02085268.1_-1<br>GGEY02074216.1_-1	100%	80.00%	0
tyrosine-protein kinase SYK-like<br><br>(SYK)	XP_022100262.1	GGEY02068414.1_+3	100%	88.18%	0
**Phosphatases**					
tyrosine-protein phosphatase non-receptor type 11-like isoform X3<br><br>(PTPN11)	XP_022100586.1	GGEY02082444.1_+3	78%	93.27%	0
tensin-2-like isoform X9<br><br>(TSN2)	XP_022079400.1	GGEY02081501.1_-3	99%	79.11%	0
phosphatidylinositol 3,4,5-trisphosphate 5-phosphatase 2B-like isoform X1<br><br>* Only SH2 domain matches to ID from both nr and uniprot matches	XP_022093863.1	GGEY02031697.1_-3	96%	76.01%	4e-165
**Other**					
beta-chimaerin-like isoform X2<br><br>(CHN2)	XP_022094840.1	GGEY02007747.1_+3<br>GGEY02007748.1_+1	100%	85.46%	0
Transforming protein cbl<br><br>(CBL)	P23092.1	GGEY02094567.1_-2	89%	64.17%	3e-115
E3 ubiquitin-protein ligase CBL-like isoform X3<br><br>(CBLC)	XP_022109070.1	GGEY02068257.1_-2	100%	86.41%	0
extensin-like isoform X1 (EXT1)<br>+<br>B-cell linker protein (BLNK)	XP_022101579.1	GGEY02003111.1_-1	95%<br>42%	65.64%<br>34.62%	5e-92<br>4e-15
filaggrin-like isoform X2 (FLG)	XP_022093373.1	GGEY02010334.1_+3	100%	89.52%	4e-140
VAV2-like isoform X3 (VAV2)	XP_022099138.1	GGEY02076094.1_+3<br>GGEY02076095.1_-2	99%	74.42%	0

phospholipase C-gamma (PLCG1)	AAR85355.1	GGEY02080031.1_+2	100%	99.68%	0
Ras and Rab interactor 2-like isoform X1<br><br>(RIN2)	XP_022091301.1	GGEY02058477.1_+1	99%	71.90%	0
Ras GTPase-activating protein 1-like<br><br>(RASA1)	XP_022105967.1	GGEY02030271.1_-2	99%	87.23%	0
signal transducer and activator of transcription 5A-like isoform X3<br><br>(STAT5A)	XP_022086666.1	GGEY02084791.1_-1	100%	89.63%	0
suppressor of cytokine signaling 2-like<br><br>(SOCS2)	XP_022103312.1	GGEY02051721.1_+2	100%	78.26%	2e-140
suppressor of cytokine signaling 5-like<br><br>(SOCS5)	XP_022093145.1	GGEY02000762.1_+3<br>GGEY02000763.1_+3	100%	95.16%	5e-129
Suppressor of cytokine signaling 6<br><br>(SOCS6)	Q5RCM6.1	GGEY02062844.1_-2	51%	58.64%	5e-57
transcription elongation factor SPT6-like<br><br>(SUPT6H)	XP_022093194.1	GGEY02051522.1_-1	100%	89.12%	0
uncharacterized protein LOC110983703	XP_022098870.1	GGEY02085023.1_+2<br>GGEY02085024.1_+2<br>GGEY02081986.1_+1<br>GGEY02080104.1_+3<br>GGEY02042805.1_+1	100%	70.55%	2e-150
uncharacterized protein LOC110991054<br><br>+<br><br>SOCS-7 (SOCS7)	XP_022111846.1<br><br>O14512.2	GGEY02008063.1_+1	100%<br><br>66%	93.41%<br><br>58.38%	3e-175<br><br>7e-67
cation channel sperm-associated protein 2-like isoform X1 (CATSPER1)<br><br>*SH2 domain that is not on cation channel match	XP_022105643.1	GGEY02034955.1_-2	100%	85.21%	0

uncharacterized protein LOC110984176 isoform X2	XP_022099764.1	GGEY02012625.1_-1	100%	95.30%	6e-95
No ID – SH2 superfamily only		GGEY02041449.1_-3			
No ID – SH2 superfamily only		GGEY02044078.1_-3			
No ID – SH2 superfamily only		GGEY02025565.1_-3			
No ID – SH2 superfamily only		GGEY02064150.1_+1			

Figure S1. Sanger sequencing results of amplified transcripts aligned to the respective transcriptome sequences

CLUSTAL O(1.2.4) multiple sequence alignment – PLCgamma

```
GGEY02080031.1_+2        TACAGCTGCTTGTGGCATGACTACGGAGTCACACTGTCTTCGATCCGGTGACTTCACTTC 60
PLCgamma_seq_result      ----------------------------------------------------TTCACTTC 8
                                                                             ********

GGEY02080031.1_+2        AGAATGGCCACCAACAGCCTCTACAAGAAGAAGCTGACGCCCCAGGAGGTGTCCAGCGTC 120
PLCgamma_seq_result      AGAATGGCCACCAACAGCCTCTACAAGAAGAAGCTGACGCCCCAGGAGGTGTCCAGCGTC 68
                         ************************************************************

GGEY02080031.1_+2        ACCAAGATGCTGAAAATGGGCACCGTCCTGACGCGCTTCTACGGCAAACGACGACCGGAA 180
PLCgamma_seq_result      ACCAAGATGCTGAAAATGGGCACCGTCCTGACGCGCTTCTACGGCAAACGACGACCGGAA 128
                         ************************************************************

GGEY02080031.1_+2        AGGAGGTCGTTTGAAATCTGCATGGAGACGCGGCAGATTCTGTGGAGGCGACAGACTGGG 240
PLCgamma_seq_result      AGGAGGTCGTTTGAAATCTGCATGGAGACGCGGCAGATTCTGTGGAGGCGACAGACTGGG 188
                         ************************************************************

GGEY02080031.1_+2        CGGACAGATGGAGCAGTTAAAATTCGTGAGATAAAAGAGATTCGTCCCGGTAAGAACTCA 300
PLCgamma_seq_result      CGGACAGACGGAGCAGTTAAAATTCGTGAGATAAAAGAGATTCGTCCCGGTAAGAACTCA 248
                         ******** ***************************************************

GGEY02080031.1_+2        CGAGACTTCGAGAGGTGGCCGGATGAAGCCAAGAAGTATGATACCTCGCTCTGTCTTGTC 360
PLCgamma_seq_result      CGAGACTTCGAGAGGTGGCCAGATGAAGCCAAGAAGTATGATACCTCGCTCTGTCTTGTC 308
                         ******************** ***************************************

GGEY02080031.1_+2        ATCTGCTATGGTGCTGAGTTCAGACTCAAGAGCTTGTCCGTCGTTGCCGGCAATGCCGAT 420
PLCgamma_seq_result      ATATGCTACGGTGCCGAGTTCAGACTCAAGAGCTTGTCCGTCGTTGCCGGCAATGCCGAT 368
                         ** ***** ***** *********************************************

GGEY02080031.1_+2        GAACGACACAAGTGGATCGTCGGCCTCAACTGGCTAGTCGAAGACCACAAAATCTCAAGT 480
PLCgamma_seq_result      GAACGACACAAGTGGATCGTCGGCCTCAACTGGCTAGTCGAAGACCACAAAATCTCAAGT 428
                         ************************************************************

GGEY02080031.1_+2        TACCCAAGCAGACTAGAATGGTGGTTACGACGGGAGTTCTACGCCATGGGAAAAACAAAG 540
PLCgamma_seq_result      TACCCAAGCAGACTAGAATGGTGGTTACGACGGGAGTTCTACGCCATGGGAAAAACAAAG 488
                         ************************************************************

GGEY02080031.1_+2        AATGATACGGTGTCACTTAGGGACATGAAGTCATTCATGCCATACGTCAACCTGAAAATG 600
PLCgamma_seq_result      AATGATACGGTGTCACTTAGGGACATGAAGTCATTCATGCCATACGTCAACCTGAAAATG 548
                         ************************************************************

GGEY02080031.1_+2        AACACAAAGGACCTTAAAGAATATTTCAATGAAGTGGATCGGTGGAACAAGCAAGAAATT 660
PLCgamma_seq_result      AACACAAAGGACCTTAAAGAATATTTCAATGAAGTGGATCGGTGGAACAAGCAAGAAATT 608
                         ************************************************************

GGEY02080031.1_+2        GGTTTTGACGGCTTTGTCCAGCTCTACCACAACCTCATCTTCCAGAGGGAGGTCGCTGAC 720
PLCgamma_seq_result      GGTTTTGACGGCTTTGTCCAGCTTTACCACAACCTCATCTTCCAGAGGGAGGTCGCTGAC 668
                         *********************** ************************************

GGEY02080031.1_+2        AGATTCAAAGAGTACATCGATGAACGAAATCTGGTGACTGTGAATGGCATGATTCGCTTC 780
PLCgamma_seq_result      AGATTCAAAGAGTACATCGATGAACGAAATCTGGTGACTGTGAATGGCATGATTCGCTTC 728
                         ************************************************************

GGEY02080031.1_+2        CTAGCCCAAGAACAAAAGGACACCACGGCTAACAATCCCATCGCAGTGAAGGCCATGATG 840
PLCgamma_seq_result      CTAGCCCAAGAACAAAAGGACACCACGGCAAACAATCCTATCGCAGTGAAGGCCATGATG 788
                         ***************************** ******** *********************

GGEY02080031.1_+2        GAATCGTTTCTGACGGACCTCGGCCGACCGTGCCAGGAGTCGGACCCCAAATTCACAGTC 900
PLCgamma_seq_result      GAATCGTTTCTGACGGACCTCGGCCGACCGTGCCAGGAGTCGGACCCCAAATTCACAGTC 848
```

```
                          ************************************************************

GGEY02080031.1_+2         CCAGAGTTTC-------------------------------------------------- 910
PLCgamma_seq_result       CCAGAGTTTCTGCTGTACTTGTTCTCGCCGGATAATGAGATCTGGGACAAGAAATTTGAT 908
                          **********

GGEY02080031.1_+2         ------------------------------------------------------------ 910
PLCgamma_seq_result       GAGATTGTGGACGACTTGGACCAACCGCTCTGTAACTACTTGATAGCTTCCTCTCACAAT 968


GGEY02080031.1_+2         ------------------------------------------------------------ 910
PLCgamma_seq_result       ACATACCTTACCGGGGATCAGATCGCTAGTGAGTCATCGTGTGAGGCGTACGTCCGATGT 1028


GGEY02080031.1_+2         ------------------------------------------------------------ 910
PLCgamma_seq_result       CTACCACAATCCTATCGCAGTGAAGGCCATGATGGAATCGTTTCTGACGGACCTCGGCCG 1088


GGEY02080031.1_+2         --------------------------------------------TGCTGTACTTGTTCTC 926
PLCgamma_seq_result       ACCGTGCCAGGAGTCGGACCCCAAATTCACAGTCCCAGAGTTTCTGCTGTACTTGTTCTC 1148
                                                                      ****************

GGEY02080031.1_+2         ACCGGATAATGAGATCTGGGACAAGAAATTTGATGAGATTGTGGACGACTTGGACCAACC 986
PLCgamma_seq_result       GCCGGATAATGAGATCTGGGACAAGAAATTTGATGAGATTGTGGACGACTTGGACCAACC 1208
                           ***********************************************************

GGEY02080031.1_+2         GCTCTGTAACTACTTGATAGCTTCCTCTCACAATACATACCTTACCGGGGATCAGATCGC 1046
PLCgamma_seq_result       GCTCTGTAACTACTTGATAGCTTCCTCTCACAATACATACCTTACCGGGGATCAGATCGC 1268
                          ************************************************************

GGEY02080031.1_+2         TAGTGAGTCATCGTGTGAGGCGTACGTCCGATGTCTACGCATGGGCTGCCGATGTCTGGA 1106
PLCgamma_seq_result       TAGTGAGTCATCGTGTGAGGCGTACGTCCGATGTCTACGCATGGGCTGCCGATGTCTGGA 1328
                          ************************************************************

GGEY02080031.1_+2         ACTTGACTGTTGGGACGGGCCGGAAGGAATGCCCATTATCTACCATGGCCTGACGCTGAC 1166
PLCgamma_seq_result       ACTTGACTGTTGGGACGGGCCGGAAGGAATGCCCATTATCTACCACGGCCTGACTCTAAC 1388
                          ********************************************* ******** ** **

GGEY02080031.1_+2         TTCAAAAATCAAATTCATGGACGTTTTGAAGACGATAAAGGAACACGCTTGGGTGCAGTC 1226
PLCgamma_seq_result       TTCAAAAATCAAATTCATGGACGTTTTGAAGACGATAAAGGAGCACGCATGGGTGCAGTC 1448
                          ****************************************** ***** ***********

GGEY02080031.1_+2         AGATTTGCCCATCATTTTATCAATCGAGAACCACTGCACACTGATACAGCAACGCAACAT 1286
PLCgamma_seq_result       AGACTTGCCCATCATTTTATCAATCGAGAACCACTGCACACTGATACAGCAACGCAACAT 1508
                          *** ********************************************************

GGEY02080031.1_+2         GGCTTCATCATTCCAGGAAGTCTTTGGAGACTCCCTTTTGACGCAGCCAGTGGATCGCGA 1346
PLCgamma_seq_result       GGCCTCATCATTCCAGGAAGTCTTTGGAGACTCCCTTTTGACGCAGCCAGTGGATCGCGA 1568
                          *** ********************************************************

GGEY02080031.1_+2         TGCCACCATACTGCCGACGGTCAACCAGCTAAGAAAGAAGATCATACTCAAGCACAAGAA 1406
PLCgamma_seq_result       TGCCACCATACTGCCGACGGTCAACCAGCTAAGAAAGAAGATCATACTCAAGCACAAGAA 1628
                          ************************************************************

GGEY02080031.1_+2         GCTGATGGGCACCAATGAAACCTTCAGCGTACCCACTGAAGAAATGTCTGGTCTTGATTT 1466
PLCgamma_seq_result       GCTGATGGGCACCAATGAAACCTTTAGCGTACCCACTGAAGAAATGTCTGGTCTTGATTT 1688
                          ************************ ***********************************

GGEY02080031.1_+2         GAGGAACTCTCTGAAGAACGGAATCCTCAAGATCGAAGACCCAATGGATAATGAATGGGT 1526
PLCgamma_seq_result       GAGGAACTCTCTGAAGAACGGAATCCTCAAGATCGAAGACCCAATGGATAATGAATGGGT 1748
                          ************************************************************

GGEY02080031.1_+2         CCCACATTACTTTGTGCTGACCACGGAGAAACTCTTCTACTGCGAGCAAACCCAAAACCT 1586
PLCgamma_seq_result       CCCACATTACTTTGTGCTGACCACGGAGAAGCTCTTCTACTGCGAGCAAACCCAAAACCT 1808
                          ****************************** *****************************
```

```
GGEY02080031.1_+2       TGTCAATCAAGATGATGACGACGACACTACTAGCCAATTGGACATGCAAGCCACGCCCAA 1646
PLCgamma_seq_result     TGTCAATCAAGATGACGATGACGACACAACTAGCCAATTGGACATGCAAGCCACGCCCAA 1868
                        *************** ** ******** ********************************

GGEY02080031.1_+2       TGATGAGCTGCACTTCTCCGAGCCCTGGTTCCACGGC----------------------- 1683
PLCgamma_seq_result     TGATGAGCTGCACTTCTCCGAGCCCTGGTTCCACGGCAAGCTGAACAGCAACAGCGACGT 1928
                        *************************************

GGEY02080031.1_+2       ------------------------------------------------------------ 1683
PLCgamma_seq_result     CACACCCAGGATGCTGGCCGAGCAACTGCTCAACCAATACCAGAAGGGAGACGGCACTTT 1988


GGEY02080031.1_+2       ------------------------------------------------------------ 1683
PLCgamma_seq_result     CCTGGTCCGAGAGAGCGAAACTTTCAAGGGGGACTACTCCTTGTCATTCTGGGCGCGAGG 2048


GGEY02080031.1_+2       ------------------------------------------------------------ 1683
PLCgamma_seq_result     CAAGGGAGCTCTTCTACTGCGAGCAACCCAAAACCTTGTCAATCAAGATGACGATGACGA 2108


GGEY02080031.1_+2       ------------------------------------------------------------ 1683
PLCgamma_seq_result     CACAACTAGCCAATTGGACATGCAAGCCACGCCCAATGATGAGCTGCACTTCTCCGAGCC 2168


GGEY02080031.1_+2       -------------AAGCTGAACAGCAACAGCGACGTCACACCCAGGATGCTGGCCGAGCA 1730
PLCgamma_seq_result     CTGGTTCCACGGCAAGCTGAACAGCAACAGCGACGTCACACCCAGGATGCTGGCCGAGCA 2228
                                     ***********************************************

GGEY02080031.1_+2       ACTGCTCAACCAATACCAGAAGGGAGACGGCACTTTCCTGGTCCGAGAGAGCGAAACTTT 1790
PLCgamma_seq_result     ACTGCTCAACCAATACCAGAAGGGAGACGGCACTTTCCTGGTCCGAGAGAGCGAAACTTT 2288
                        ************************************************************

GGEY02080031.1_+2       CAAGGGGGACTACTCCTTGTCATTCTGGGCGCGAGGCAAGGTGAACCACTGCCGCATCCG 1850
PLCgamma_seq_result     CAAGGGGGACTACTCCTTGTCATTCTGGGCGCGAGGCAAGGTGAACCACTGCCGCATCCG 2348
                        ************************************************************

GGEY02080031.1_+2       GTACAAGCTAGACCAGAGCCGGCCCAAGTACTTCTTGGTGGAGCACACGTGCTTCGATAG 1910
PLCgamma_seq_result     GTACAAGCTAGACCAGAGCCGGCCCAAGTACTTCTTAGTGGAGCACACGTGCTTCGATAG 2408
                        ************************************ ***********************

GGEY02080031.1_+2       TCTCTACAGCCTCATCTCCCACTACCGCCAGTGCCCCTTGCGTAGCCGGGGGCTCGAGCT 1970
PLCgamma_seq_result     TCTCTACAGCCTCATCTCCCACTACCGCCAGTGCCCCTTGCGTAGCCGGGGGCTCGAGCT 2468
                        ************************************************************

GGEY02080031.1_+2       CCTACTGACGGAGCCCGTGCCACAACCCCTCAGCCACGAGGGAAAAGATTGGTTTCATAA 2030
PLCgamma_seq_result     CCTACTGACAGAGCCCGTGCCTCAACCCCTCAGCCACGAGGGAAAAGATTGGTTTCATAA 2528
                        ********* *********** **************************************

GGEY02080031.1_+2       GAAGTTGAGCCGGCCTCAGGCAGAAGAAATGCTGAAACGAGTCCACCAGGATGGCTCGTT 2090
PLCgamma_seq_result     GAAGTTGAGCCGGCCTCAGGCAGAAGAAATGCTGAAACGAGTGCACCAGGATGGCTCGTT 2588
                        ****************************************** *****************

GGEY02080031.1_+2       CCTGGTCAGAAAGAGAGAGCAGGGAGATGACTCCTACGCCATATCATTCAGGGCGGAGGG 2150
PLCgamma_seq_result     CCTGGTCAGGAAGAGAGAACAGGGAGATGACTCCTACGCCATATCATTCAGGGCGGAGGG 2648
                        ********* ******** *****************************************

GGEY02080031.1_+2       TAAGATCAAGCACTGCCGCATCAACCAGGAGGGCCGGCTCTTTGCCATCGGCAATGCCCA 2210
PLCgamma_seq_result     TAAGATCAAGCACTGCCGCATCAACCAGGAGGGCCGGCTCTTTGCCATCGGCAACGCCCA 2708
                        ****************************************************** *****

GGEY02080031.1_+2       CTTTGAGAGCATCGTGGAGTTGGTCTCCTACTACGAGAAGTTCCCGCTCTATCGCAAGAT 2270
PLCgamma_seq_result     TTTTGAGAGCATCGTGGAGTTGGTCTCCTACTACGAGAAGTTCCCGCTCTACCGCAAGAT 2768
                         ************************************************** ********

GGEY02080031.1_+2       GAAGCTCAAGTACCCGGTCAACCAAGAGATCGTGGACCGACTCGGAGGGGCAGCAGATGA 2330
PLCgamma_seq_result     GAAGCTCAAGTACCCGGTCAACCAGGAGATCGTGGACCGACTAGGAGGGGCAGCAGATGA 2828
```

```
                          *********************** **************** ****************

GGEY02080031.1_+2         GAATTCCTTGTATGGTAACCCGGAACTCTACATGGACCCAAACCAGTTTGTACCAAAGGT 2390
PLCgamma_seq_result       GAATTCCTTGTATGGTAACCCGGAACTCTACATGGACCCAAACCAGTTTGTACCAAAGGT 2888
                          ************************************************************

GGEY02080031.1_+2         GACAGTGAAAGCGCTGTATGACTACAAGGCCCAACGAGACGATGAGTTGA---------- 2440
PLCgamma_seq_result       GACGGTGAAAGCACTGTATGACTACAAGGCCCAACGAGACGATGAGTTGACATTCTGCAA 2948
                          *** ******** *************************************

GGEY02080031.1_+2         ------------------------------------------------------------ 2440
PLCgamma_seq_result       GCACGCCATCATCACCAATGTAGATAAGCAAGACCTTGGCTGGTGGAAAGGAGACTATGG 3008


GGEY02080031.1_+2         ------------------------------------------------------------ 2440
PLCgamma_seq_result       CGGCAANAAGAACATGTGGTTCCCCTCCACTACGTGGAANANACCCAACCAAATGACAAC 3068


GGEY02080031.1_+2         ------------------------------------------------------------ 2440
PLCgamma_seq_result       AGTCCCGAGTCAACGCTACTAGGCANCCGGGCAGCAGATGAGAATTCCTTGTATGGTAAC 3128


GGEY02080031.1_+2         ------------------------------------------------------------ 2440
PLCgamma_seq_result       CCGGAACTCTACATGGACCCAAACCAGTTTGTACCAAAGGTGACGGTGAAAGCACTGTAT 3188


GGEY02080031.1_+2         -------------------------------CATTCTGCAAGCACGCCATCATCACCAAT 2469
PLCgamma_seq_result       GACTACAAGGCCCAACGAGACGATGAGTTGACATTCTGCAAGCACGCCATCATCACCAAT 3248
                                                         *****************************

GGEY02080031.1_+2         GTAGATAAGCAAGACCTTGGCTGGTGGAAAGGAGACTACGGCGGCAAGAAGAACATGTGG 2529
PLCgamma_seq_result       GTAGATAAGCAAGACCTTGGCTGGTGGAAAGGAGACTATGGCGGCAAGAAGAACATGTGG 3308
                          ************************************** *********************

GGEY02080031.1_+2         TTCCCCTCCAACTACGTGGAGGAGACCCAACCAAACGACAACAGTCCCGAGTCAACGCTA 2589
PLCgamma_seq_result       TTCCCCTCCAACTACGTGGAAGAGACCCAACCAAATGACAACAGTCCCGAGTCAACGCTA 3368
                          ******************** ************** ************************

GGEY02080031.1_+2         CTAGGCAACCTGCAGAAGGGGGCTATTGACATCCGAAGATGCGCTGTTGAGACGCTACCC 2649
PLCgamma_seq_result       CTAGGCAACCTGCAGAAGGGGGCTATTGACATCCGAAGATGCGCTGTTGAGACGCTACCC 3428
                          ************************************************************

GGEY02080031.1_+2         GCAAGTCGATCCACACACCCCAACGTCTTCAGAATCTCCCCTCTGAACAACCGGGGGGCC 2709
PLCgamma_seq_result       GCAAGTCGATCCACACACCCCAATGTCTTCAGAATCTCCCCTCTGAACAACCGGGGGGCC 3488
                          *********************** ************************************

GGEY02080031.1_+2         ATCGACCTGGCAGCATCTTCGACCGAAGACCTAAGCGACTGGATTCAGACGATAGAGGAT 2769
PLCgamma_seq_result       ATCGACCTGGCGGCTTCTTCGACCGAAGACCTAAGCGACTGGATTCAGACGATAGAGGAT 3548
                          *********** ** *********************************************

GGEY02080031.1_+2         GCCTCACTCAAGGCTGAGGCCAGGCGATTGGAAGAGACCAAGCATGAACGCAAGATGCGC 2829
PLCgamma_seq_result       GCCTCACTTAAAGCTGAGGCCAGGCGATTGGAAGAGACCAAGCATGAACGCAAGATGCGC 3608
                          ******** ** ************************************************

GGEY02080031.1_+2         ATCCCCAAGGAAT----------------------------------------------- 2842
PLCgamma_seq_result       ATCGCCAAGGAATTCTCCGATCTCATCGTGTACTGCAGATCAGTACCTTTCCGTGAAGAT 3668
                          *** *********

GGEY02080031.1_+2         ----------------------TCATGTCCTCATTCCCAGAGACCAAGGTGGAGAGGTAC 2880
PLCgamma_seq_result       AACATTCCCGGCAAATACTACGACATGTCCTCATTCCCAGAGACCAAGGTGGAGAGGTAC 3728
                                                 *************************************

GGEY02080031.1_+2         CTTACAGCGATCAAATCCAAGCTACTACTGTGCTACAACCAGCACCAGGTCAGTAGGACG 2940
PLCgamma_seq_result       CTTACAGCAATCAAATCCAAGCTACTACTGTGCTACAACCAGCACCAGGTCAGTAGGACG 3788
                          ******** ***************************************************
```

```
GGEY02080031.1_+2       TATCCGAAGGGCCAGCGCTTTGATTCCTCCAACTACGACCCGGTCCCAATCTGGAACATC 3000
PLCgamma_seq_result     TATCCGAAGGGCCAGCGCTTTGATTCCTCCAACTACGACCCGGTCCCAATCTGGAACATC 3848
                        ************************************************************

GGEY02080031.1_+2       GGCACACAGATGGTGTCTCTCAACTACCAGACACCAGACCGCTATATGCAAATCAACGAA 3060
PLCgamma_seq_result     GGCACACAGATGGTGTCTCTCAACTACCAGACACCAGACCGCTATATGCAAATCAACGAA 3908
                        ************************************************************

GGEY02080031.1_+2       GGCTTCTTCGCCTTGAACGGCCGCTGTGGTTACGTCTTGACGCCCCCCTGCATGAGAGAC 3120
PLCgamma_seq_result     GGCTTCTTCGCCTTGAACGGCCGCTGCGGTTACGTCTTGACGCCCCCCTGCATGAGAGAC 3968
                        ************************** *********************************

GGEY02080031.1_+2       CCCAACTTTGACCCCTACGATCCCCGCACCATGCAAGGAGTGGAACCCA----------- 3169
PLCgamma_seq_result     CCCAACTTTGACCCCTACGATCCCCGCACCATGCAAGGAGTGGAACCCATTCATCTCAAT 4028
                        *************************************************

GGEY02080031.1_+2       ------------------------------------------------------------ 3169
PLCgamma_seq_result     ATTACGATTTTGGCAGCTCGTCACCTTGAGAAGACCGGACGGAGTATCGCTAGTCCCTTT 4088

GGEY02080031.1_+2       ------------------------------------------------------------ 3169
PLCgamma_seq_result     GTTGAAGTGGAGATCATTGGTTACGTCTTGACGCCCCCCTGCATGAGAGACCCCAACTTT 4148

GGEY02080031.1_+2       ----------------------------------------TTCATCTCAATATTACGATT 3189
PLCgamma_seq_result     GACCCCTACGATCCCCGCACCATGCAAGGAGTGGAACCCATTCATCTCAATATTACGATT 4208
                                                                ********************

GGEY02080031.1_+2       TTGGCAGCTCGTCACCTTGAGAAGACCGGACGGAGTATCGCTAGTCCCTTTGTTGAAGTG 3249
PLCgamma_seq_result     TTGGCAGCTCGTCACCTTGAGAAGACCGGACGGAGTATCGCTAGTCCCTTTGTTGAAGTG 4268
                        ************************************************************

GGEY02080031.1_+2       GAGATCATCGGTGTGGAACGGGACAGCACCCAGAAGTACAAGACGCAGACAATAGCTGAC 3309
PLCgamma_seq_result     GAGATCATTGGTGTGGAACGGGACAGCACCCAGAAGTACAAGACGCAGACAATAGCTGAC 4328
                        ******** ***************************************************

GGEY02080031.1_+2       AACGGCTTCAACCCGATATTTAACGAGCGCTGCGAGTTTGACGTCGTCAACAAGGACCTG 3369
PLCgamma_seq_result     AACGGCTTCAACCCGATATTTAACGAGCGCTGCGAGTTTGACGTCGTCAACAAGGACCTG 4388
                        ************************************************************

GGEY02080031.1_+2       GCCTTCATCCGCTTCGTCTTGCAAGACGAAGACGTATTCGGTGACCCCAACTTCCTGGGT 3429
PLCgamma_seq_result     GCCTTCATCCGCTTCGTCTTGCAAGACGAAGACGTATTCGGTGACCCCAACTTCCTGGGT 4448
                        ************************************************************

GGEY02080031.1_+2       CACAGGACGCTGCCCCTTCGGGCAATCAGAACAGGGTATCGCACAGTGCCGTTAATGAAT 3489
PLCgamma_seq_result     CACAGGACGCTGCCCCTTCGGGCAATCAGAACAGGGTATCGCACAGTGCCATTAATGAAT 4508
                        ************************************************** *********

GGEY02080031.1_+2       GGCCACTCGGAGTTCTTAGAGCTGGCGTCACTGCTCATCCATGTAGAATACAGGGTTATC 3549
PLCgamma_seq_result     GGCCACTCGGAGTTCTTAGAGCTGGCGTCACTGCTCATCCATGTAGAATACAGGGTTATC 4568
                        ************************************************************

GGEY02080031.1_+2       GGGGAATGCGGAGACAACGACCTGTACGCCTCCATCCAGTGCCTGCGCGACCAGACCGAG 3609
PLCgamma_seq_result     GGGGAATGCGGAGACAACGACCTGTACGCCTCCATCCAGTGCCTGCGCGACCAGACCGAG 4628
                        ************************************************************

GGEY02080031.1_+2       AAACTGACCAAACAGATCGGGGACATGGAGCTAACCAATGCCGCGTCCAGACGGCCGGAC 3669
PLCgamma_seq_result     AAACTGACCAAACAGATCGGGGACATGGAGCTGACCAATGCCGCGTCCAGACGGCCGGAC 4688
                        ******************************** ***************************

GGEY02080031.1_+2       GAAGCCTACCACGCCAAGTCGATAGAGCTGGAGAGGTGCCAGGACGCGCTCTTCAGACTC 3729
PLCgamma_seq_result     GAAGCCTACCACGCCAAGTCGATAGAGCTGGAGCGCTGCCAGGACGCGCTCTTCAGACTC 4748
                        ********************************* * ************************

GGEY02080031.1_+2       CAGGAACAGAGAAGCGCCAAACTGAAGAAGACTAACAGCAACAGTGCATGA 3780
PLCgamma_seq_result     CAGGAACAGAGACNCCCCCAAAANNGNGAA--------------------- 4778
```

```
                         ************  * ** **       *
```

CLUSTAL O(1.2.4) multiple sequence alignment

```
EXT_BLNK                 GCTTCTGCCGATCAAGGAGTGCGCCCACAAAAAAAACATTAGCGCTATCAGCGGAAGTTT 60
EXT_BLNK_sequencing      ----------------------------------------------------CGGANTTT 8
                                                                              * * ***

EXT_BLNK                 GGCGCTGCCACCTTCAGGCAAGTCTTGCCCGCGTCTCCGGCCAAGATTAGAGGGCTGGTC 120
EXT_BLNK_sequencing      GGCGCCGCCACCTTCAGGCAAGTCTTGCCCGCGTCTCCGGCCAAGATTAGAGGGGTGGTC 68
                         ***** ************************************************ *****

EXT_BLNK                 ATATGGTGCTGGATAAGAAGTACTGCCGTGTCGAAAACCTGTTCGTTGTCCTTGTAGGTG 180
EXT_BLNK_sequencing      ATGTGATGCTGGATAAGAAGTACTGCCGTGTCAAAAACCTGTTCGTTGTCCTTGTAGGTG 128
                         ** ** ************************** ***************************

EXT_BLNK                 CCCAGAGCGTACTTGCCGTCGTCCCGCAGTCGGATGTGCAGGTTCCTGACCTTGTTGTTG 240
EXT_BLNK_sequencing      CCCAGAGCGTACTTGCCGTCGTCCCGCAGTCGGATGTGCAGGTTCCTGACCTTGTTGTTG 188
                         ************************************************************

EXT_BLNK                 AACCAGAGAGACAGCGAGTAGGGGATGTTGTCTCCTCCTTTGGTACTGTTCCGGACAACA 300
EXT_BLNK_sequencing      AACCAGAGAGACAGCGAGTAGGGGATGTTGTCTCCTCCTTTGGTACTGTTCCGGACAACA 248
                         ************************************************************

EXT_BLNK                 AATGCACCATCCATTTTGGCTTGTTTGAGGATCTGCACTGCTTCGTCACGGCTTAGATCA 360
EXT_BLNK_sequencing      AATGCACCATCCATTTTGGCTTGTTTGAGGATCTGCACTGCTTCGTCACGGCTTAGATCA 308
                         ************************************************************

EXT_BLNK                 GCATCATACCATGGAAAGCCCGTCATTTCATTCCTCGTGGGAGGTTGAGGCATGGTTCTT 420
EXT_BLNK_sequencing      GCATCATACCATGGAAAGCCCGTCATTTCATTCCTCGTGGGAGGTTGAGGCATGGTTCTT 368
                         ************************************************************

EXT_BLNK                 GGATCAGGAGGGGGAGCTGGTGGCGCCTCTGCGGGTATCTGCTTAGGAAGGTCTGGCACC 480
EXT_BLNK_sequencing      GGGTCAGGAGGGGGAGCTGGTGGCGCCTCTGCGGGTATCTGCTTAGGAAGGTCTGGCACC 428
                         ** *********************************************************

EXT_BLNK                 CTGCTGCTGTGTGGAGTGAACTTCTTGATTGGTTTGGCAGGTTCCTTGGTGGGAATCGGG 540
EXT_BLNK_sequencing      CTGCTGCTGTGAGGAGTGAACTTCTTGATTGGTTTGGCAGGTTCCTTGGTGGGAATCGGG 488
                         *********** ************************************************

EXT_BLNK                 GGAGTTGGCTTGTGTTCGGGGCTCGTCGGAGGCTCTGCGGGTAACCGAGATCTAGGAGTT 600
EXT_BLNK_sequencing      GGAGTTGGCTTGTGCTCGGGGCTCGTCGGAAGCTCTGCGGGTAACCGAGATCTAGGAGTT 548
                         ************** *************** *****************************

EXT_BLNK                 GGTCGTGGAGGTGCATGTGCCGACGACGATGCTGGGGTGTGTGGAAGGCTGGGCACGTTT 660
EXT_BLNK_sequencing      GGTCGTGGAGGTGCATGTGCCGACCANNATGCTGGTGTTTGTGGAAGGCTGGGCACGTTT 608
                         ************************ *  ******* ** *********************

EXT_BLNK                 GCTGCCTGATTGTTAGCCTCCGCTATGGCCGAGTTCAGCCACGACTGAACGGGTTTGTTT 720
EXT_BLNK_sequencing      GCTGCCTGATTGTTAGCCTCCGCTATGGCCGAGTTCAGCCACGACTGAACGGGTTTGTTT 668
                         ************************************************************

EXT_BLNK                 GTGTTCGGCTTGGCTGACAACTCCGGTTTATTCCCTAGCGGCCTGGGAAGCGGCGGCGGC 780
EXT_BLNK_sequencing      GTGTTCGGCTTGGCTGACAACTCCGGTTTATTCCCTAGCGGCCTGGGAAGCGGCGGCGGC 728
                         ************************************************************

EXT_BLNK                 CGCATGGACTCTGTGTCTTCGGCTGCCCCCTGAAACTGCTGCAACCTTCTGC-------- 832
EXT_BLNK_sequencing      CGCATGGACTCTGTGTCTTCGGCTGCCCCCTGAAACTGCTNAAACCTTTTCNGCAAAAAG 788
                         ****************************************  ****** *

EXT_BLNK                 -----        832
EXT_BLNK_sequencing      ACAAA        793
```

CLUSTAL O(1.2.4) multiple sequence alignment

```
SFK1_sequencing      ----------------------------------------------------GGNACAAG     8
SFK1                 CTTCTTGGCCAACATCGTAGCCGCCATGGGGTGCATCAACAGCAAAGAGGATGGCACAAG     60
                                                                     ** *****

SFK1_sequencing      CTACCGAGCCGATACCAGAAACGACAGCAACGACATGGGCAGCCCGGGCATGAAGATAAC     68
SFK1                 CTACCGCGCCGATACCAGAAACGACAGCAACGACATGGGCAGCCCGGGCATGAAGATAAC     120
                     ****** *****************************************************

SFK1_sequencing      AACTCCGTCACCGACCATTGCCACCGGAGGTGTCACCGATTTCAGGGGAAACAGTCAGAT     128
SFK1                 AACTCCGTCACCGACCATTGCCACCGGAGGTGTCACCGATTTCAGGGGAAACAGTCAGAT     180
                     ************************************************************

SFK1_sequencing      GTTCATCCCTACCCCTGCTCAACCTAAACCCAGCAAACCAGTACTGAAATACGTTGCCAT     188
SFK1                 GTTCATCCCTACCCCTGCTCAACCTAAACCCAGCAAACCAGTACTGAAATACGTTGCCAT     240
                     ************************************************************

SFK1_sequencing      TTACGATTACGAGGCGCGAACAGGAGACGACTTGAGCTTTCTGAAAGGAGAAACACTAGA     248
SFK1                 TTACGATTACGAGGCGCGAACAGGAGACGACTTGAGCTTTCTGAAAGGAGAAACACTAGA     300
                     ************************************************************

SFK1_sequencing      TATAACGAATAACAACGATGGTGATTGGTGGCTGGCAAGGTCAACGAAGACTTTACAAGA     308
SFK1                 TATAACGAATAACAACGATGGTGATTGGTGGCTGGCAAGGTCAACGAAGACTTTACAAGA     360
                     ************************************************************

SFK1_sequencing      AGGTTACGTCCCCAGCAACTACATCGCACCTGTCAAGAGCATCAGTGCTGAAGAGTGGTA     368
SFK1                 AGGTTACGTCCCCAGCAACTACATCGCACCTGTCAAGAGCATCAGTGCTGAAGAGTGGTA     420
                     ************************************************************

SFK1_sequencing      CTTTGGTCGTATCGGTCGCAAGGAAGCAGAGAAGAAGCTTGTAATGCCTGGTGTCGAGCG     428
SFK1                 CTTTGGTCGTATCGGTCGCAAGGAAGCAGAGAAGAAGCTTGTAATGCCTGGTGTCGAGCG     480
                     ************************************************************

SFK1_sequencing      AGGCATGTTCATTGTGAGAGACGGTGAAGCCACACCAGGTACTTTTTCACTTTCTGTTCG     488
SFK1                 AGGCATGTTCATTGTGAGAGACGGTGAAGCCACACCAGGTACTTTTTCACTTTCTGTTCG     540
                     ************************************************************

SFK1_sequencing      TGACTACGACCCAGTTAAAGGGGACCACGTGAAACACTACAAGATCCGAAAGCTAGACAA     548
SFK1                 TGACTACGACCCGGTCAAAGGCGACCACGTGAAACACTACAAGATCCGAAAGCTAGACAA     600
                     ************ ** ***** **************************************

SFK1_sequencing      CGAGGCGGGATTCTACATAGCCATGCGCAGCCCCTTTCCGGTTCTGGCTGAGCTAGTCAA     608
SFK1                 CGAGGCGGGATTCTACATAGCCATGCGCAGCCCCTTTCCTGTTCTGGCTGAGCTAGTCAA     660
                     *************************************** ********************

SFK1_sequencing      ACATTACCAACAGGTGGCTGATGGACTGTGCATTAAGCTGACCTTCCCATGCCCCAAGGA     668
SFK1                 ACATTACCAACAGGTGGCTGATGGACTGTGCATTAAGCTGACCTTCCCTTGCCCCAAGGA     720
                     ************************************************ ***********

SFK1_sequencing      GAACCCCAACACCGTCAGTCTGGGCAGAGATGCTTGGGAAATCCCCCGCACCTCCCTGAC     728
SFK1                 GAACCCCAACACTGTCAGCCTGGGCAGAGATGCTTGGGAAATCCCCCGCACTTCCCTGAC     780
                     ************ ***** ******************************** ********

SFK1_sequencing      GCTGGAGAGCAAGCTTGGGGCCGGCCAGTTTGGAGAAGTCTGGAAAGGTACTTGGAATGG     788
SFK1                 GCTGGAAAGCAAGCTTGGGGCCGGCCAGTTTGGAGAAGTCTGGAAAGGTACTTGGAATGG     840
                     ****** *****************************************************

SFK1_sequencing      GAAGACCCCAGTGGCCATCAAGACCCTCAAGAAGGGCACTATGACTCCCACGGCCTTCCT     848
SFK1                 GAAGACCCCAGTGGCCATCAAGACCCTCAAGAAGGGCACCATGACTCCCACGGCCTTCCT     900
                     *************************************** ********************

SFK1_sequencing      GGCAGAAGCCAACATCATGAAGAAACTCCGCCATCCAAAACTCTGTCAGCTCTACGCCGT     908
SFK1                 GGCAGAAGCCAACATCATGA----------------------------------------     920
                     ********************
```

```
SFK1_sequencing      CTGCTCCGATAAGGAACCATCTACATCGTGGCTGAGTTGATGTGCAACGGCAGTCTTTTG     968
SFK1                 ------------------------------------------------------------     920

SFK1_sequencing      GACTTCTTGAAAGACGGCGAAGGCCGCAACCTGAAGTTGCCGGAACTGGTTGACATGGGN     1028
SFK1                 ------------------------------------------------------------     920

SFK1_sequencing      AGCTCAGATTGCATCTGGCATGGCCTTCCTCGAGTCCTTGANCTTTCAGACCCTCANGAA     1088
SFK1                 ------------------------------------------------------------     920

SFK1_sequencing      GGGCACNNTGACTCCCACAGGCCTTCCTGGCAGAAGCCAACATCATGAAGAAACTCCGCC     1148
SFK1                 ------------------------------------------------AGAAACTCCGCC     932
                                                                     ************

SFK1_sequencing      ATCCAAAACTCTGTCAGCTCTACGCCGTCTGCTCCGATAAGGAACCCATCTACATCGTGG     1208
SFK1                 ATCCAAAACTCTGTCAGCTCTACGCCGTCTGCTCCGATAAGGAACCCATCTACATCGTGG     992
                     ************************************************************

SFK1_sequencing      CTGAGTTGATGTGCAACGGCAGTCTTTTGGACTTCTTGAAAGACGGCGAAGGCCGCAACC     1268
SFK1                 CTGAGTTGATGTGCAACGGCAGTCTTTTGGACTTCTTGAAAGACGGCGAAGGCCGCAACC     1052
                     ************************************************************

SFK1_sequencing      TGAAGTTGCCGGAGCTGGTTGACATGGGAGCTCAGATTGCATCTGGCATGGCCTTCCTCG     1328
SFK1                 TGAAGTTGCCGGAGCTGGTTGACATGGGAGCTCAGATTGCATCTGGCATGGCCTTCCTCG     1112
                     ************************************************************

SFK1_sequencing      AGTCCATGAACTACGTCCATCGTGATCTGGCCGCTAGGAACGTCCTGGTGGGAGAGGGCA     1388
SFK1                 AGTCCATGAACTACGTCCATCGTGATCTGGCCGCTAGGAACGTCCTGGTGGGAGAGGGCA     1172
                     ************************************************************

SFK1_sequencing      ACATCGTCAAGGTGGCTGATTTTGGGCTGGCCAGGATGATTGAAGATACCGAGTACACTG     1448
SFK1                 ACATCGTCAAGGTGGCTGATTTTGGGCTGGCCAGGATGATTGAAGATACCGAGTACACTG     1232
                     ************************************************************

SFK1_sequencing      CTAGACAAGGTGCCAAGTTTCCAATCAAATGGACAGCCCCAGAGGCGGCCATGTACGGAC     1508
SFK1                 CTAGACAAGGTGCCAAGTTTCCAATCAAATGGACAGCCCCAGAGGCGGCCATGTACGGAC     1292
                     ************************************************************

SFK1_sequencing      GCTTCACCATCAAATCCGATGTCTGGTCCTTCGGTGTTCTACTGACTGAGTTGGTCACAC     1568
SFK1                 GCTTCACCATCAAATCCGATGTCTGGTCCTTCGGTGTTCTACTGACTGAGTTGGTCACAC     1352
                     ************************************************************

SFK1_sequencing      ACGGACGTATACCATACCCAGGTATGATGAACATGGAGGTCTTGGACCAGGTGGAGCACG     1628
SFK1                 ACGGACGTATACCATACCCAGGTATGATGAACATGGAGGTCTTGGACCAGGTGGAGCACG     1412
                     ************************************************************

SFK1_sequencing      GCTACCGCATGCCCAAGATGGCCAATTGCCCGGACACCCTCTACGAACTCATGCAGAAGT     1688
SFK1                 GCTACCGCATGCCCAAGATGGCCAATTGCCCGGACACCCTCTACGAACTCATGCAGAAGT     1472
                     ************************************************************

SFK1_sequencing      GTTGGGACAAGGATCCTGCCGCCAGACACACCTTCGAGTTCCTGCATTCCTACCTGGATG     1748
SFK1                 GTTGGGACAAGGATCCTGCCGCCAGACACACCTTCGAGTTCCTGCATTCCTACTTGGATG     1532
                     ***************************************************** ******

SFK1_sequencing      ACTACTTCGTCGCGACGGAGCCCAACTACAAGGAAGCGGAGTAAACTCGGTGGTGAGAAC     1808
SFK1                 ACTACTTCGTCGCGACGGAGCCCAACTACAAGGAAGCGGAGTAAACTCGGTGGTGAGAAA     1592
                     ***********************************************************

SFK1_sequencing      AAGTCAAAGTGTNTAGTTCGNCGCGGGGGGNG     1840
SFK1                 AAGTCAAAGTGTGTGATGTCCGTGGTG-----     1619
                     ************ *  *       * *

CLUSTAL O(1.2.4) multiple sequence alignment
```

```
SFK3_sequencing      ---------------------------------------------NNNNNNGN-----NN     10
SFK3                 TAAGATGGCGGCCAAACTCGTGGTTCTACTGATACTGGATCTCTGAGGCGTGATAGAAGT     60
                                                                        *

SFK3_sequencing      NNNNCCGNNGNGANNNGNNNT----CNNGGTCTGTNGNGCGACCATGCCGTCACTGTGCC     66
SFK3                 CGTCCAGGAAAGAGTGGAGGAACTCGAAGGTTGGTCGCTTCTCAGGGACAGCATCCCAGC     120
                         * *    **   *           ***  ** *     *   * *  **      *

SFK3_sequencing      CTTCCTNAGNGNTGGANACGTTGTGACTTGGACCGAGCCCACA-GTCGCCGGCNACTCTG     125
SFK3                 ATTGCTTGGTGATATCG----TA-GAGCTGGTCCGGGCATTCTGGAGGCTTGGGCATCCG     175
                      ** **  * * *        *  **  *** *** **   *  *  **  *    ** *

SFK3_sequencing      GAGCCTTCAGTACACAACNNACGCATAATTCCGGTGACTTCTTTAAGGCCAACNCGCCCT     185
SFK3                 ATAGCCACGGGAGACCTGGTCTAACACCTCCCGGTTCACCATGCCTGGATAAGGCACCCT     235
                         *  * * * **             * *****      *    **  **  * ****

SFK3_sequencing      ANNTGACTGGNAANNCTCGTAAATAAGAATTCCGAAGGACCAGACATCGGACTTGATGGT     245
SFK3                 CCCTTTAGTGATGACCTCGTAAATAAGAATTCCGAAGGACCAGACATCGGACTTGATGGT     295
                        *     *     *********************************************

SFK3_sequencing      GAACTTGCCGAAGTTTATGGCCTCAGGAGCGGTCCATTTGATGGGCATCTTTGCACCTTC     305
SFK3                 GAACTTGCCGAAGTTTATGGCCTCAGGAGCGGTCCATTTGATGGGCATCTTTGCACCTTC     355
                     ************************************************************

SFK3_sequencing      CTTTGCGATGTACTCATCTTCGATGATACGCGAGAGTCCAAAGTCGGCAACTTTGCAATT     365
SFK3                 TTTTGCGATGTACTCATCTTCGATGATACGCGAGAGTCCAAAGTCGGCAACTTTGCAATT     415
                      ***********************************************************

SFK3_sequencing      TCTGAGGTCTCCCACCAAGATGTTCCTGGCTGCCAGGTCACGATGGACGAATCGCTGTGA     425
SFK3                 TCTGAGGTCTCCCACCAAGATGTTCCTTGCTGCCAGGTCACGATGGACGAATCGCTGTGA     475
                     *************************** ********************************

SFK3_sequencing      TTCCAGGTACGCCATGCCGTCTGCAACCTGTGCCGTGATATCGATGAGCTCGGGTTCCCG     485
SFK3                 TTCCAGGTACGCCATGCCGTCTGCAACCTGTGCCGTGATATCGATGAGCTCGGGTTCCCG     535
                     ************************************************************

SFK3_sequencing      TAGTTGCTTCCCCTCTCCCTCGTGCAGGTAATCCAGCAGACAGCCGTTCGTCATCAGCTC     545
SFK3                 TAGTTGCTTCCCCTCTCCCTCGTGCAGGTAATCCAGCAGACAGCCGTTCGTCATCAGCTC     595
                     ************************************************************

SFK3_sequencing      CGTCACTATGTAGATTGGCTCTCCCTCTGAACAGACGGCGAGAAGAGCCACCAGTTTCGG     605
SFK3                 CGTCACTATGTAGATTGGCTCTCCCTCTGAACAGACGGCGAGAAGAGCCACCAGTTTCGG     655
                     ************************************************************

SFK3_sequencing      ATGCCGCAGTTTCTTCATGATGTTGGCTTCTTGAAGGAAGGCCGTGGGATCCATGGCGCC     665
SFK3                 ATGCCGCAGTTTCTTCATGATGTTGGCTTCTTGAAGGAAGGCCGTGGGATCCATGGCGCC     715
                     ************************************************************

SFK3_sequencing      TTCCTTCAACGTCTTCACTGCAACTCGCACGGTTCCATTCCATGTACCTTCCCAAACCTC     725
SFK3                 TTCCTTCAACGTCTTCACTGCAACTCGCACGGTTCCATTCCATGTACCTTCCCAAACCTC     775
                     ************************************************************

SFK3_sequencing      GCCGAATTGTCCATTGCCCAGTTTGCGTTCCAGCTTGATGGAGCGACGCGGAATCTCCCA     785
SFK3                 GCCGAATTGTCCATTGCCCAGTTTGCGTTCCAGCTTGATGGAGCGACGCGGAATCTCCCA     835
                     ************************************************************

SFK3_sequencing      GACGTCTTTGCTGATCTCAAACATGACGGGTTTCTGGCGTGGGCAAGGGTTCAGTAGACG     845
SFK3                 GACGTCTTTGCTAATCTCAAACATGACGGGTTTCTGGCGTGGGCAAGGGTTCAGTAGACG     895
                     ************ ***********************************************

SFK3_sequencing      GCAGACCAGTCCGTCTGCTTGGCTTGTATAATGGTTGACCAAATCATGTAGCGTATGGAA     905
SFK3                 GCAGACCAGTCCGTCTGCTTGGCTTGTATAATGGTTGACCAAATCATGTAGCGTATGGAA     955
                     ************************************************************
```

```
SFK3_sequencing      AGTAATGCGTGTGCTGATGTAGTAACCACCATTGTCCAGAGTCCTTATGCGGTAATGTTT     965
SFK3                 AGTAATGCGTGTGCTGATGTAGTAACCACCATTGTCCAGAGTCCTTATGCGGTAATGTTT     1015
                     ************************************************************

SFK3_sequencing      GACGTTAAAGCCTCTAGCGTCGTCATGATCCAAGACGGATAACGAGTAGGCACCTGGACT     1025
SFK3                 GACGTTAAAGCCTCTAGCGTCGTCATGATCCAGCACGGATAACGAGTAGGCA--------     1067
                     ********************************  ******************

SFK3_sequencing      CGTTTCGCTGCCCCTAATGAGGAATGTCCCCCGGANNNNNNNNCNTANGGAAAGTAATGC     1085
SFK3                 ------------------------------------------------------------     1067

SFK3_sequencing      GTGTGCTGATGTAGTAACCACCATTGTCCAGAGTCCTTATGCGGTAATGTTTGACGTTAA     1145
SFK3                 ------------------------------------------------------------     1067

SFK3_sequencing      AGCCTCTAGCGTCGTCATGATCCAAGACGGATAACGAGTAGGCACCTGGACTCGTTTCGC     1205
SFK3                 -----------------------------------------CTACCTGGACTCGTTTCGC     1086
                                                                *****************

SFK3_sequencing      TGCCCCTAATGAGGAATGTCCCCCGGGAGTTTCCCGCCAGCTGAAGCTGCTTCTCCGCTT     1265
SFK3                 TGCCCCTAATGAGGAATGTCCCCCGGGAGTTTCCCGCCAGCTGAAGCTGCTTCTCCGCTT     1146
                     ************************************************************

SFK3_sequencing      CCTTTCTCGTCATCTTCTCGAAGAACCAGTCTTCCGCCTGAAGTACATCCGCCGGTTCGA     1325
SFK3                 CCTTTCTCGTCATCTTCTCGAAGAACCAGTCTTCCGCCTGAAGTACATCCGCCGGTTCGA     1206
                     ************************************************************

SFK3_sequencing      TGTAGTTTCTCGGCACATAACCTTCTCGCTTCGTCAGCATTGAGGTAGCCAACCACCAGT     1385
SFK3                 TGTAGTTTCTCGGCACATAACCTTCTCGCTTCGTCAGCATTGAGGTAGCCAACCACCAGT     1266
                     ************************************************************

SFK3_sequencing      TGGGGTCGGTTTTATTTGTGATAATGAGTATCTCCCCCTTTCGGAAAGTGAGATCGTCTG     1445
SFK3                 TGGGGTCGGTTTTATTTGTGATAATGAGTATCTCCCCCTTTCGGAAAGTGAGATCGTCTG     1326
                     ************************************************************

SFK3_sequencing      CTGTCCTGGCTTCATAGTCATACAGTGCTTTGTAACGCTCACCAGCAGGTCTTGTACTAG     1505
SFK3                 CTGTCCTGGCTTCATAGTCATACAGTGCTTTGTAACGCTCACCAGCAGGTCTTGTACTAG     1386
                     ************************************************************

SFK3_sequencing      GCGAGTGCATGATTCCCATCTGATCATTACCCACGCCAGCTGAGATCATAACCCCAACGG     1565
SFK3                 GCGAGTGCATGATTCCCATCTGATCATTACCCACGCCAGCTGAGATCATAACCCCAACGG     1446
                     ************************************************************

SFK3_sequencing      ATCCAGCACCGCCTCCTCCAACGTCTCCACCAACATGGTTCTGCCGGTCCTGATCGCCTC     1625
SFK3                 ATCCAGCACCGCCTCCTCCAACGTCTCCACCAACATGGTTCTGCCGGTCCTGATCGCCTC     1506
                     ************************************************************

SFK3_sequencing      CTGCCCCACTACTTCCAATCCCAGCACCGCTACTCGAAGCAGCATTTACGGAGTCCTTAC     1685
SFK3                 CTGCCCCACTACTTCCAATCCCAGCACCGCTACTCGAAGCAGCATTTACGGAGTCCTTAC     1566
                     ************************************************************

SFK3_sequencing      GAGCCTTGCCCTTCTTGGGCTTTTTGTCTTTGTCCTTCCGGTCCTTCCTGTCCCGGCCAA     1745
SFK3                 GAGCCTTGCCCTTCTTGGGCTTTTTGTCTTTGTCCTTCCGGTCCTTCCTGTCCCGGCCAA     1626
                     ************************************************************

SFK3_sequencing      GTTTAGCTGAGGATACGGTGATGGTTGAGGTGCTGTGGTCATAGCCGTCTGGGCCGTCAA     1805
SFK3                 GTTTAGCTGAGGATACGGTGATGGTTGAGGTGCTGTGGTCATAGCCGTCTGGGCCGTCAA     1686
                     ************************************************************

SFK3_sequencing      GTCCGTTACTATGGGCGCTTTCCTTGTTTCCCATGCTGGGTCTGTGGTAGGGACACGCCT     1865
SFK3                 GTCCGTTACTATGGGCGCTTTCCTTGTTTCCCATGCTGGGTCTGTGGTAGGGACACGCCC     1746
                     *********************************************************** 

SFK3_sequencing      ANNTGAAATGGGTAAA 1881
SFK3                 TAACTGACTGTCA--- 1759
```

```
                           * **

CLUSTAL O(1.2.4) multiple sequence alignment


SH2WW_sequencing      -----------------------------NNNNNNNNNATCTGGCNNCTGACGTATCAAT    31
SH2WW                 GGACCAATACTCCGCTTGACAATGGCCAGGCCGACAGATCTGCGACTGAACGTATCAAAT    60
                                                                 *             ***

SH2WW_sequencing      CAGAGAGAATCCAATATGCCTCCAGTACCAGCAACTTCATTCCGACCATATGGTGGAGAT    91
SH2WW                 CAGAGAGAATCCAATATGCCTCCAGTACCAGCAACTTCATTCCGACCATATGGTGGAGAT    120
                      ************************************************************

SH2WW_sequencing      TGTGTGGATGCCCAGGGCCTTGACTTCTACTATGGAAAAATTGAAAATACACTGGTTGAG    151
SH2WW                 TGTGTGGATGCCCAGGGCCTTGACTTCTACTATGGAAAAATTGAAAATACACTGGTTGAG    180
                      ************************************************************

SH2WW_sequencing      GAACTCTTAAGACCCCACATGGAGTCTAGAGGGTCTTTTCTTCTCCGAGACAGCAACACT    211
SH2WW                 GAACTCTTAAGACCCCACATGGAGTCTAGAGGGTCTTTTCTTCTCCGAGACAGCAACACT    240
                      ************************************************************

SH2WW_sequencing      GAACCTGGTGTTATCACAATATCTGTGGTAGATAACCCCCGCCACATCTACCACTTCAAG    271
SH2WW                 GAACCTGGTGTTATCACAATATCTGTGGTAGATAACCCCCGCCACATCTACCACTTCAAG    300
                      ************************************************************

SH2WW_sequencing      ATCAACTGCAGGGGTCCCAACGGCGTGTATGAAGTCACTGATAAACTCCGTTTTGGCTCC    331
SH2WW                 ATCAACTGCAGGGGTCCCAACGGCGTGTATGAAGTCACTGATAAACTCCGTTTTGGCTCC    360
                      ************************************************************

SH2WW_sequencing      CTGGAAGATCTCATCAAGTTTTTTCACTTCAATGACGTCCCAAGCAGAAACATCGCTGAC    391
SH2WW                 CTGGAAGATCTCATCAAGTTTTTTCACTTCAATGACGTCCCAAGCAGAAACATCGCTGAC    420
                      ************************************************************

SH2WW_sequencing      CTGAAACTTGTGAATCCAATCCGCTTCAGAAGTACCCCCCGCAGCCCACAGCGGAGCCCT    451
SH2WW                 CTGAAACTTGTGAATCCAATCCGCTTCAGAAGTACCCCCCGCAGCCCACAGCGGAGCCCT    480
                      ************************************************************

SH2WW_sequencing      CCACGGAGCCCACCCACAAGCCCTCCCATGGAACTAAGATCACCGTCTCGCCCACCCCGT    511
SH2WW                 CCACGGAGCCCGCCCAGAAGCCCTCCCATGGAACTAAGATCACCGTCTCGCCCACCCCGT    540
                      *********** **** *******************************************

SH2WW_sequencing      CCACCAAGAATTATAAGTCCCCCAGCGACAAGGGATCAAGACAGATTTCTGGATTCAAGG    571
SH2WW                 CCACCAAGAATTATAAGTCCCCCAGCGACAAGGGATCAAGACAGATTTCTGGATTCAAGG    600
                      ************************************************************

SH2WW_sequencing      GCCCCAGTTCCCATCCCCACACCAGGAACAGAACCGATATACATCTATCATGACCACGTT    631
SH2WW                 GCCCCAGTTCCCATCCCCACACCAGGAACAGAACCGATATACATCTATCATGACCACGTT    660
                      ************************************************************

SH2WW_sequencing      TACTGTCGGGCTGAGGATCTCCAGAAATCACGGATAGATGAGATCATGGAACTGTGCAAG    691
SH2WW                 TACTGTCGGGCTGAGGATCTCCAGAAATCACGGATAGATGAGATCATGGAACTGTGCAAG    720
                      ************************************************************

SH2WW_sequencing      TTGTCAGATCAGAAGTACAAGAAGTGCACCTGTGGTTTGTACATGGAACAGTCCATACTG    751
SH2WW                 TTGTCAGATCAGAAGTACAAGAAGTGCACCTGTGGTTTGTACATGGAACAGTCCATACTG    780
                      ************************************************************

SH2WW_sequencing      GTAGAAGATTGGATGATGCATATCGATAGACCAGCTGGTGATCAGGCAGAAAGTGGGCGT    811
SH2WW                 GTAGAAGATTGGATGATGCATATCGATAGACCAGCTGGTGATCAGGCAGAAAGTGGGCGT    840
                      ************************************************************

SH2WW_sequencing      GTCTTCTTTGTGAATTCAGTCAGTAATCTTACACAGTGGGAGTTACCAGAGAGAGTTATG    871
SH2WW                 GTCTTCTTTGTGAATTCAGTCAGTAATCTTACACAGTGGGAGTTACCAGAGAGAGTTATG    900
                      ************************************************************
```

```
SH2WW_sequencing      TATCAACTTAGCCAACAGTATCCTGAAAAATACAGCTTTGTGCAGAAACTTTTGCAGGAG    931
SH2WW                 TATCAACTTAGCCAACAGTATCCTGAAAAATACAGCTTTGTGCAGAAACTTTTGCAGGAA    960
                      *********************************************************** 

SH2WW_sequencing      GGCAGTAATCTATAATGGTTGNNCNGNAAAAAAAAANTTTNNNNAAATAA    981
SH2WW                 GGCAGTAATCTTTAAATGGTTGATCAGTTACGAACATTTGT---------    1001
                      *********** ***  * *         *  ** * **
```
